# Hepatic circadian clock oscillators and nuclear receptors integrate microbiome-derived signals

**DOI:** 10.1038/srep20127

**Published:** 2016-02-16

**Authors:** Alexandra Montagner, Agata Korecka, Arnaud Polizzi, Yannick Lippi, Yuna Blum, Cécile Canlet, Marie Tremblay-Franco, Amandine Gautier-Stein, Rémy Burcelin, Yi-Chun Yen, Hyunsoo Shawn Je, Al-Asmakh Maha, Gilles Mithieux, Velmurugesan Arulampalam, Sandrine Lagarrigue, Hervé Guillou, Sven Pettersson, Walter Wahli

**Affiliations:** 1 INRA ToxAlim, UMR1331, Chemin de Tournefeuille, Toulouse Cedex, France; 2 Department of Microbiology, Tumor and Cell Biology (MTC), Karolinska Institutet, Stockholm, Sweden; 3 Department of Medicine, Division of Cardiology, UCLA, Los Angeles, USA; 4 Institut National de la Santé et de la Recherche Médicale, U855, Lyon, France; 5 Institut des Maladies Métaboliques et Cardiovasculaires, Hôpital Rangueil, Toulouse Cedex, France; 6 Molecular Neurophysiology Laboratory, Signature Program in Neuroscience and Behavioral Disorders, Duke-NUS Graduate Medical School, Singapore, Singapore; 7 Department of Physiology, Yong Loo Lin School of Medicine, National University of Singapore, Singapore; 8 Department of Health Sciences, College of Arts and Sciences, Qatar University, Daha, Qatar; 9 INRA, UMR1348 Pegase, Saint-Gilles, France; 10 Agrocampus Ouest, UMR1348 Pegase, France; 11 Rennes; Université Européenne de Bretagne, France; 12 Lee Kong Chian School of Medicine, Nanyang Technological University Singapore, Singapore; 13 SCELSE microbiome centre, Nanyang Technological University, Singapore, Singapore; 14 Center for Integrative Genomics, University of Lausanne, Le Genopode, Lausanne, Switzerland

## Abstract

The liver is a key organ of metabolic homeostasis with functions that oscillate in response to food intake. Although liver and gut microbiome crosstalk has been reported, microbiome-mediated effects on peripheral circadian clocks and their output genes are less well known. Here, we report that germ-free (GF) mice display altered daily oscillation of clock gene expression with a concomitant change in the expression of clock output regulators. Mice exposed to microbes typically exhibit characterized activities of nuclear receptors, some of which (PPARα, LXRβ) regulate specific liver gene expression networks, but these activities are profoundly changed in GF mice. These alterations in microbiome-sensitive gene expression patterns are associated with daily alterations in lipid, glucose, and xenobiotic metabolism, protein turnover, and redox balance, as revealed by hepatic metabolome analyses. Moreover, at the systemic level, daily changes in the abundance of biomarkers such as HDL cholesterol, free fatty acids, FGF21, bilirubin, and lactate depend on the microbiome. Altogether, our results indicate that the microbiome is required for integration of liver clock oscillations that tune output activators and their effectors, thereby regulating metabolic gene expression for optimal liver function.

The mammalian body is a complex ecosystem that consists of a eukaryotic core and a myriad of microorganisms, including the gut microbiome (microbial communities and their metabolites). Together, they form a holobiont, whose parts live in symbiosis with a high capacity to adapt to changing environmental conditions^1^,^2^.

Indigenous microbe populations have co-evolved with their hosts to meet mutually beneficial biological needs. In particular, they support the host by promoting digestion and absorption of food ingredients, such as complex dietary carbohydrates and lipids, thus maximizing the energy harvest from ingested food^3^.

Intestine and liver are derived from the primitive gut, which develops into these two complex metabolically mature organs^4^. Once differentiated, these organs maintain a direct communication via the portal vein and the bile duct. The venous blood from the intestine is the main supplier of absorbed gut products, including nutrients and microbial metabolites. The hepatic output, in response to incoming metabolites and nutrients, comprises the production of bile acid. Bile acid is discharged via the bile duct into the intestine to tune intestinal host metabolism and modulate the abundance and distribution of various microbiome components^5^. Such interactions may suggest a possible link between certain metabolic liver diseases and the composition and roles of the gut microbiome. In fact, perturbations of the gut microbiome have been associated with common liver disorders, such as non-alcoholic fatty liver disease, non-alcoholic steatohepatitis, alcoholic liver disease, and liver cirrhosis^5^,^6^.

Almost all living organisms are exposed to the light–dark cycle, and their activity–rest cycles and feeding behaviours have been adapted to this oscillation. This 24-hour rhythm is driven by a biological circadian clock whose functions are well documented not only in the suprachiasmatic nucleus of the brain but also in liver, muscle, and intestine, key organs for energy homeostasis^7^. Repeated and lasting misalignment between internal clocks and activity, including shift work, and frequent cross-time zone travel, contributes to the development of metabolic diseases. Consistently, disruption of clock circuitry genes has been associated with metabolic disturbances^8^, alteration of xenobiotic detoxification^9^, bone homeostasis^10^, and epileptic seizures^11^.

Recently, a new link between the intestinal peripheral clock, the microbiome, and systemic metabolism has been unveiled^12^. Antibiotic-induced depletion of the gut microbiome results in alteration of circadian gene expression in enterocytes and of rhythmic corticosterone production in the gut, with an ensuing hypercorticosterolism that causes hyperglycaemia, insulin resistance, and increased triglycerides and free fatty acids in plasma. Taken together, these data suggest that the gut microbiome is an integral component of mechanisms that synchronize the intestinal epithelial clock. The intestinal microbiome undergoes diurnal oscillation in composition and function, as well^13^,^14^.

Despite the newly acquired information about microbiome–host bidirectional interactions, little is known about the microbiome–liver axis. Although the gut microbiome has been implicated in regulation of liver metabolism, including the hepatic molecular clock^14^, much remains to be explored on its effects on the daily rhythms of hepatic physiological patterns. It is well established that feeding rhythm drives circadian transcriptional regulation by the liver clock^15^; however, how the microbiome is involved in this process remains elusive. The data herein demonstrate an altered clock expression pattern in mice lacking a microbiome, which results in shifts in clock-controlled gene expression. In particular, the activity of nuclear receptors activated by fatty acids and cholesterol is profoundly affected. Altogether, these data indicate that the microbiome contributes not only to harvesting energy from nutrients but also to collecting or producing signals that are essential to controlling the hepatic clock genes and their effectors such as nuclear receptors.

## Results

### Gut microbiome is required for both liver core clock tuning and clock-controlled functions

**Figure 1:**
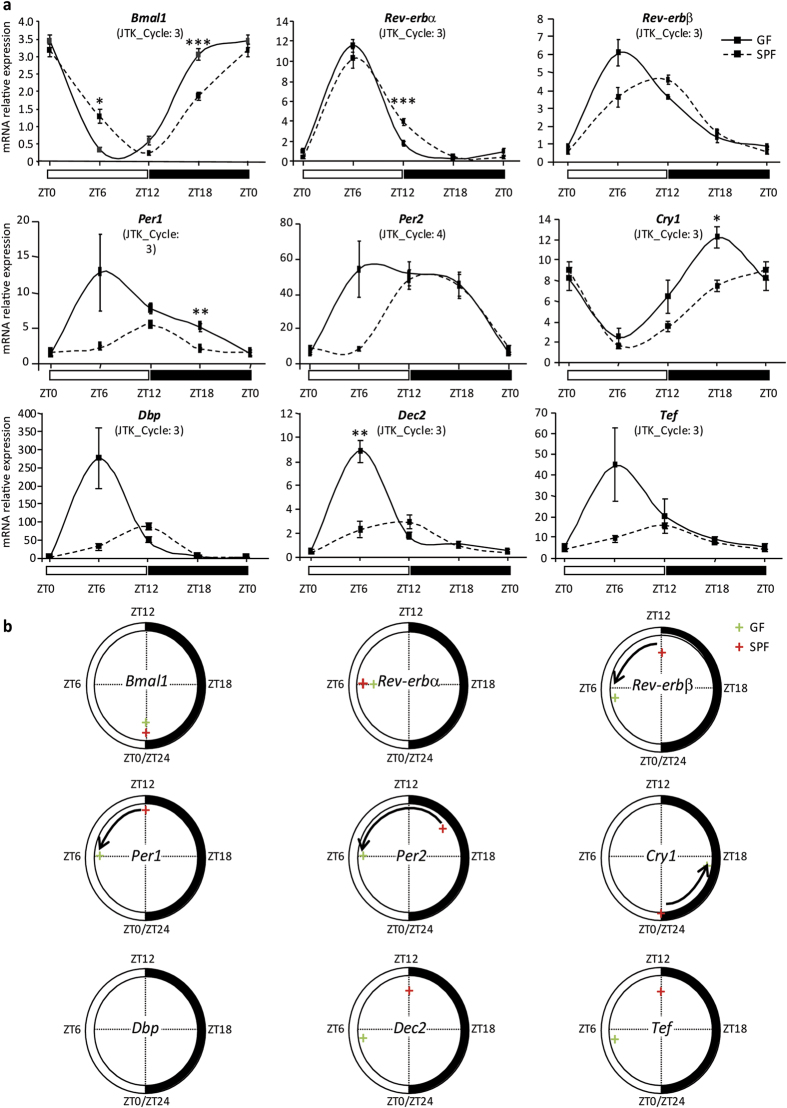
Gut microbiome influences hepatic core clock and clock-controlled gene expression. (**a**) Hepatic mRNA levels were measured at ZT0, ZT6, ZT12, and ZT18 by real-time quantitative PCR. Data presented are the mean ± SEM of relative expression values measured in germ-free (GF; black line) and specific pathogen–free (SPF; black dotted line) mice (n = 5 animals/group/ZT time point). Statistical analyses were performed with Student’s t-test at each time point between SPF and GF mice. In case of unequal variances between the two group samples, the Wehch’s two sample t-test was used. *P* values were corrected for multiple testing using BH procedure and FDR < 5% threshold is considered for significant difference. ***FDR < 0.005, **FDR < 0.01, *FDR < 0.05. (**b**) Graphical representation of acrophase (mode of expression) of hepatic core clock genes estimated by JTK_Cycle analysis for SPF (red cross) and GF (green cross) mice. The arrow indicates the shift for each gene expression tested between SPF and GF condition.

Based on the recently established link between the peripheral clock in intestinal epithelial cells and the microbiome^12^, we hypothesized that the microbiome also impacts liver physiology possibly via the liver clock core, which consists of two interlocked feedback loops that comprise several clock genes^7^. Liver and blood samples from C57Bl/6 male mice maintained in germ-free (GF) or specific pathogen–free (SPF) conditions were collected around the clock to address the question. Analysis of gene expression from liver samples showed that *Bmal1, Rev-erbα, Rev-erbβ, Per1, Per2, and Cry1* displayed significantly different mRNA expression patterns in GF mice compared to SPF mice as demonstrated by analysis with the JTK_Cycle algorithm^16^ (Fig. 1a,b; see Supplementary Tables S1 and S2 online). *Cry1* gene expression was shifted toward the dark period whereas *Rev-erbβ*, *Per1* and *Per2* expression was shifted toward the light period (Fig. 1b). Moreover, Bmal1, Rev-erbα, Per1, Cry1 mRNA levels were higher at some specific time points in GF mice compared to SPF mice. Therefore, we next assessed whether the altered expression pattern of the hepatic clock core components also affected genes encoding the output transcription factors, namely the PAR bZip factors Dbp and Tef, and the basic helix–loop–helix factor Dec2 (Bhlhb42) (Fig. 1a and see Supplementary Tables S1 and S2 online). The expression pattern of these liver clock output effectors was disturbed in GF mice as their rhythmic period of gene expression was modified in GF compared to SPF mice. Interestingly, analysis of gene expression correlation between the hepatic core clock genes and their effectors showed that their gene expression pattern was globally conserved in liver of GF compared to SPF mice, suggesting that the hepatic clock in GF mice is functional but with a perturbed expression pattern. (see Supplementary Fig. S1 online). Monitoring of GF and SPF mice in an open field for 24 hours showed no major difference in their light/dark phase locomotor activity patterns suggesting that differences observed in hepatic clock gene expression were not due to a switch in light/dark activity (see Supplementary Fig. S2 online). Collectively, these observations implied a microbiome-mediated regulation of the expression patterns of clock-connected genes and output regulators they control.

**Figure 2:**
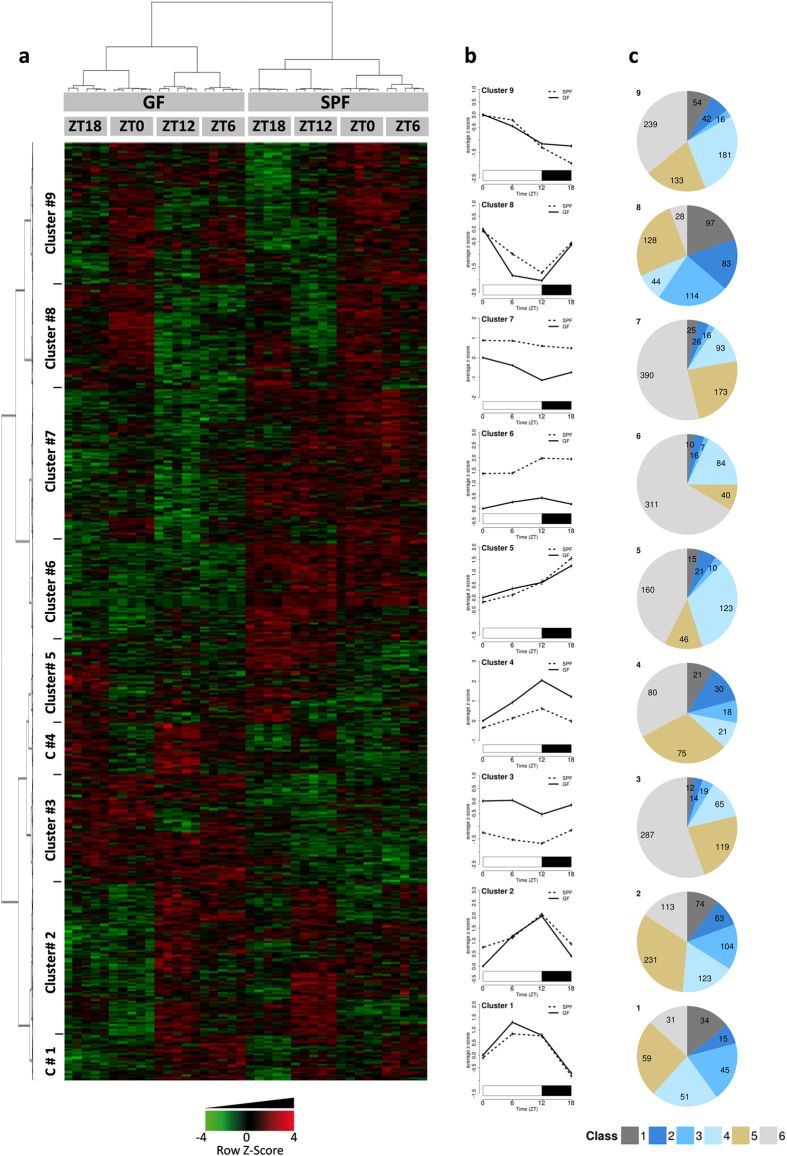
Gut microbiome affects the hepatic transcriptome and the daily hepatic gene expression. (**a**) Hepatic transcriptome was analysed at ZT0, ZT6, ZT12, and ZT18 in GF and SPF mice using Affymetrix Mouse Gene 2.0 ST arrays. Each group comprised 5 mice (a total of 40 animals). A model was fitted using the limma lmFit function. Probes with FDR < 1% (BH procedure) were considered significantly regulated. A hierarchical clustering was obtained from individual’s expression values of 4429 significantly regulated ProbeSets overall comparisons using 1-Pearson correlation coefficient as distance and the Ward’s criterion for agglomeration. Red and green colors presented in the heatmap indicate values above and below the mean centred and scaled expression values, respectively. Black indicates values close to the mean. The ProbeSets clustering and individuals clustering are illustrated on left panel dendrogram and top panel dendrogram, respectively. (**b**) Gene expression profiles of the 9 ProbeSets clusters. Mean expression values centred and scaled (average Z-score) are plotted for each sanitary status along the Zeitgeber time (ZT) (GF, black line and SPF, black dotted line). Data are scaled so that values for SPF mice at ZT0 equal zero (reference group). Error bars represent the 95% confidence interval (n = 5 animals/group/ZT time point). (**c**) Rhythmicity analysis (JTK_Cycle) on ProbeSets. For each ProbeSets cluster, the rhythmicity classes distribution is illustrated as pie charts. ProbeSets are categorized into 6 classes according to rhythmicity significance and parameters (period and phase lag) results regarding SPF and GF mice. Class 1 (dark grey): significant rhythmic ProbeSets with identical rhythmicity parameters for both SPF and GF mice; Class 2 (dark blue): significant ProbeSets in both SPF and GF mice but with a phase lag for GF mice; Class 3 (blue): significant rhythmic ProbeSets in both SPF and GF mice but with a different period for GF mice; Class 4 (light blue): significant rhythmic ProbeSets in SPF but not in GF mice; Class 5 (beige): significant rhythmic ProbeSets in GF but not in SPF mice; Class 6 (light grey): no significant rhythmic ProbeSets neither in SPF nor in GF mice.

By performing microarray gene expression analysis on liver samples, we identified more than 4000 transcripts as significantly differentially expressed (FDR < 1%) between GF and SPF. Strikingly, the hierarchical clustering of the individual gene expression values on these significantly regulated transcripts provided evidence of distinct transcriptomic trajectories of GF and SPF livers (Fig. 2a). All 40 GF and SPF animals included in the experiments exhibited a remarkable consistency in regard to both their expression pattern (GF versus SPF) and the four ZT time points of sample collection. Based on the clustering results illustrated on the heatmap, it is obvious that GF and SPF mice showed marked differences in their gene expression oscillation profile over 24 hours.

Analysis of the transcripts with altered daily - we use the term “daily” rather than “circadian” because the study was performed using standard light/dark conditions - regulation in the absence of a microbiome revealed nine major gene clusters for which a gene ontology (GO) enrichment analysis was performed, which define hepatic GO biological processes that are dependent on gut microbiome and/or daily rhythm (see Supplementary Table S3 online). For each gene cluster, the median gene expression profile was drawn along ZT time points in SPF and GF mice, defining 9 specific gene expression patterns (Fig. 2b). A rhythmicity analysis (JTK_Cycle) was performed on all transcripts and the results were summarized as pie charts for each 9 gene clusters (Fig. 2c and see Supplementary Excel File S1 online). The results revealed that in all clusters, genes with a significant (FDR < 5%) daily rhythmic expression in the SPF liver samples were affected by microbiome depletion (referred to as classes 2, 3, 4 and 5) (Fig. 2c). Especially, the majority of genes in clusters 1, 2, 4, 5, 8 and 9 saw their rhythmic expression strongly affected by the absence of microbiome, whereas genes in clusters 3, 6 and 7 mainly displayed a very large difference in their mRNA expression levels between GF and SPF. Moreover, gene expression in clusters 3, 4, 6, and 7 was increased (clusters 3 and 4) or decreased (clusters 6 and 7) in GF mice compared to SPF mice (Fig. 2b). In the two latter showing a particularly low expression in GF compared to SPF mice independently of the ZT, the GO enriched terms are related to oxidative stress, lipid metabolic processes, long-chain fatty acid transport, and organic acid metabolic processes (clusters 6 and 7). Those that showed an overall higher expression in GF versus SPF mice showed enriched biological processes in activation of the immune response, protein catabolism, carboxylic acid catabolic processes, and blood coagulation (clusters 3 and 4). In clusters 1, 2, 5, 8, and 9, the microbiome modified the rhythmic gene expression pattern and the related GO functions at specific time points. Interestingly, GO terms enriched in GF mice at ZT6 comprise cellular response to starvation, autophagic vacuole assembly, and negative regulation of intestinal cholesterol absorption (cluster 1). The absence of a microbiome affected most of the hepatic functions including diverse metabolic processes, inflammation, and haemostasis. Altogether, these results underscore the profound impact that the microbiome has on global hepatic gene expression.

To further our understanding of the liver gene expression profiling observed in the absence of a microbiome, we established expression correlation networks of liver clock genes. Because the liver clock genes affected in the GF mice are known transcription factors, we assumed that many of their downstream target genes also would be affected. To address this point, we selected the top 50 most correlated genes in the SPF condition among them those with an expression significantly correlated (adjusted P value < 0.05) either positively or negatively with each of these transcription factors (see Supplementary Fig. S3 online). The interactions among the 50 genes themselves are also depicted in the figure. We observed that under the GF condition, the correlation networks of Cry1, Rev-erbα, Rorγ, and Per2 were highly affected because out of the expression of 50 genes significantly correlated (P < 0.05) in SPF (indicated in magenta), the expression of only 8–15 (also in magenta in GF) remained significantly correlated under this condition. In addition, the density of these four networks was highly influenced in the absence of a microbiome, implying that the correlation between the expression levels of the genes belonging to these networks was significantly altered. The correlation network for the six other liver clock members (Clock, Bmal1, Rev-erbβ, Per1, Cry2, Rorα) was less affected (see Supplementary Fig. S4 online). Altogether, these results underscore the profound impact that the microbiome has on global hepatic gene expression with significant modulations of the liver core clock genes and their downstream targets.

### The microbiome regulates the activity of key hepatic transcription factors involved in metabolism and detoxification

**Figure 3:**
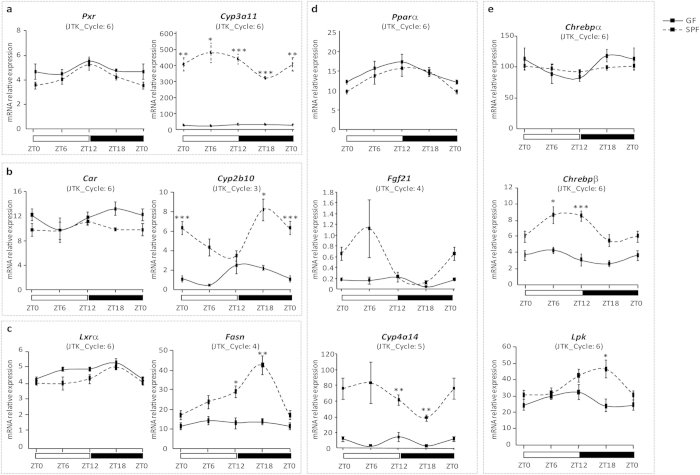
Daily expression of fatty acid, xenobiotic, sterol, and glucose sensors and their target genes under SPF and GF conditions. Hepatic mRNA levels of transcription factors and their respective target genes: (**a**) *Pxr* and *Cyp3a11*, (**b**) *Car* and *Cyp2b10*, (**c**) *Lxrα* and *Fasn*, (**d**) *Pparα, Fgf21*, and *Cyp4a14*, and (**e**) *Chrebp α* and *β* and *Lpk* measured at ZT0, ZT6, ZT12, and ZT18 by real-time quantitative PCR. Data are the mean ± SEM of relative expression values measured in GF and SPF mice (n = 5 animals/group/ZT time point). Statistical analyses were performed with Student’s t-test at each time point between SPF and GF mice. In case of unequal variances between the two samples, the Wehch’s two sample t-test was used. *P* values were corrected for multiple testing using BH procedure and FDR < 5% threshold is considered for significant difference. ***FDR < 0.005, **FDR < 0.01, *FDR < 0.05.

**Figure 4:**
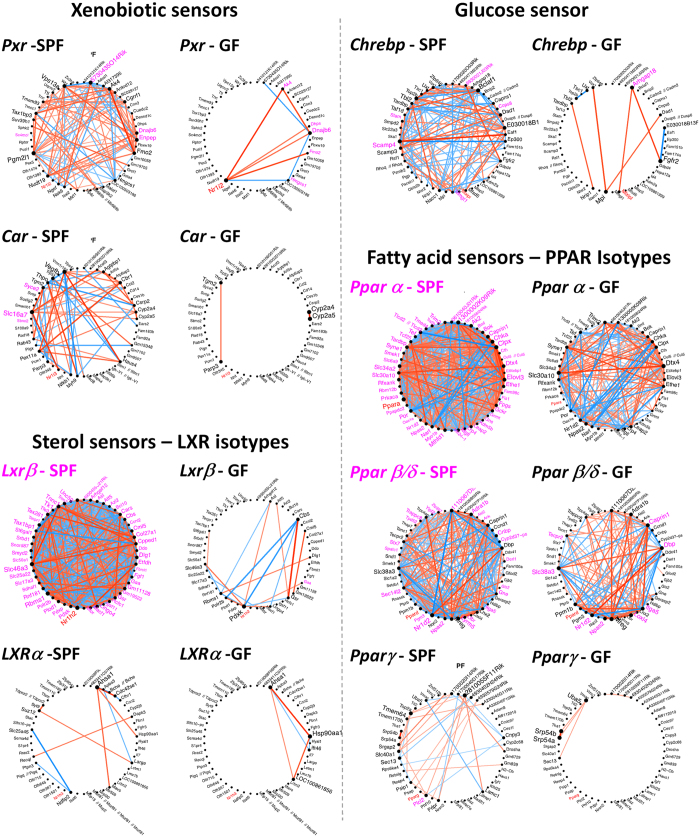
Gut microbiome strongly impacts hepatic gene expression correlation networks of *LXRβ* and *PPARα.* Networks of the 50 genes showing the highest absolute correlation with each gene of interest, *Pxr, Car, Lxrβ and α, Chrebp, and Pparα*, *β*, *and* γ (red node) under SPF condition (n = 20 mice) are presented as circle plots. The edges corresponding to significant correlations are represented (Bonferroni-adjusted *P* value < 5%). Another network circle plot based on these 51 genes is then presented in GF mice. Magenta nodes correspond to genes significantly correlated with the gene of interest. The thickness of the edges reflects the absolute correlation, and red/blue were used for positive/negative correlations, respectively. The size of each node indicates the connectivity in the circle plots.

Because the microarray data implied an insufficiency in network connectivity in xenobiotic, lipid, and glucose metabolism in GF mice not exposed to the microbiome, we next assessed whether key hepatic regulatory mediators also were affected. Among these, nuclear receptors are transcription factors whose activity is controlled by ligands. They are crucial for the regulation of many metabolic and detoxification genes in the liver, where several of them present a daily expression pattern^17^. If microbiome depletion had no or little effect on expression levels of PXR and CAR (detoxification) or LXRα and PPARα (lipid metabolism), the expression of their target genes was deeply influenced (Fig. 3a–d). Indeed, *Cyp3a11* and *Cyp2b10*, target genes of PXR and CAR, respectively, encode a xenobiotic and a drug/steroid-metabolizing enzyme. These two genes, as well as *Fasn* (Fatty acid synthesis) and *Cyp4a14* (Fatty acid hydroxylation), which are target genes of LXRα and PPARα, respectively, showed a very low expression in the liver of GF mice, with no or very dampened daily oscillations (Fig. 3a–d). This result also applies to FGF21, another target gene of PPARα, one that encodes a circulating FGF belonging to a group of proteins believed to function as classic peptide hormones^18^ (Fig. 3d). FGF21 is a nutritionally regulated hormone that induces a broad range of beneficial metabolic effects and emerges as a key player in a mechanism coordinating behaviour, growth, and metabolism during periods of reduced dietary protein intake^19^. These findings suggest a possible shortage of endogenous ligands or of other factor/s required to activate the nuclear receptors and that the gut microbiome and its metabolites are essential components in activating these receptors. Analysis of the gene expression correlation networks for PPARs, PXR, CAR, and LXRs, as was done for the clock genes, revealed alterations in these networks in the absence of the microbiome, which were particularly severe for LXRβ and PPARα (Fig. 4 and see Supplementary Figs S5 and S6 online).

Carbohydrate metabolism counts among the major functions of the liver, and the Carbohydrate-responsive element-binding proteins α and β (ChREBPα and β) are activated in response to high glucose concentrations in this organ^20^,^21^. ChREBPα and β were first identified by their ability to bind the carbohydrate response element (ChRE) of the liver pyruvate kinase (LPK) gene. Our analysis showed that they are affected differently in GF compared to SPF mice (Fig. 3e). Although the expression of ChREBPα was not significantly affected, ChREBPβ expression was strongly reduced. The expression of *Lpk* was also modified in the GF mice, particularly during the dark phase, when it was much lower than in SPF mice (Fig. 3e). The gene expression correlation network of ChREBP, similar to those of the other transcription factors analysed, was also strongly affected under the GF condition (Fig. 4).

Altogether, these gene expression profiling results are consistent with a robust effect of the gut microbiome on liver clock oscillation, influencing many key hepatic functions through modification of the transcriptional activity of several major liver factors acting as fatty acid, endobiotic, xenobiotic, sterol, and glucose sensors.

### Intestinal gluconeogenesis does not interfere with liver metabolic oscillations

**Figure 5:**
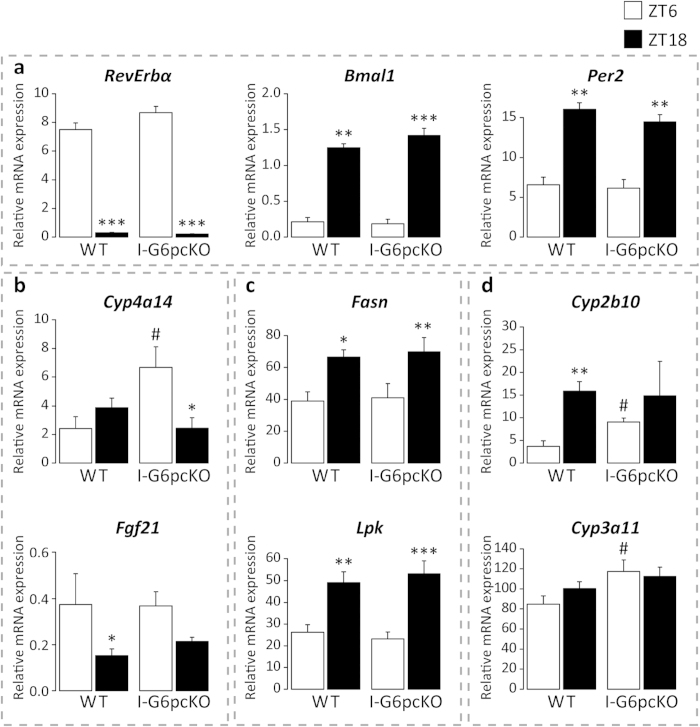
Intestinal gluconeogenesis does not impact liver clock, PPARα, LXR, ChREBP, CAR, and PXR activities. Hepatic mRNA levels of (**a**) *Rev-erbα, Bmal1, Per2*, (**b**) *Cyp4a14* and *Fgf21*, (**c**) *Fasn* and *Lpk*, and (**d**) *Cyp2b10* and *Cyp3a11* were measured at ZT6 and ZT18 by real-time quantitative PCR. Data are the mean ± SEM of values measured in wild-type mice (WT) and in mice with intestine-specific deletion of G6Pase (I-G6PcKO) (n = 6 animals/genotype/ZT time point). Statistical analyses were performed with Student’s t-test. In case of unequal variances between the two samples, the Wehch’s two sample t-test was used. *P* values were corrected for multiple testing using BH procedure and FDR < 5% threshold is considered for significant difference. * represents difference between ZT time points within a genotype; ***FDR < 0.005, **FDR < 0.01, *FDR < 0.05. ^#^represents difference between genotype for a ZT time point; ^#^FDR < 0.05.

Gut microbes generate short-chain fatty acids (SCFAs) by fermentation of soluble dietary fibres. Among these SCFAs, propionate is a substrate of intestinal gluconeogenesis (IGN) whose regulation is necessary for the metabolic benefits associated with fibre and SCFAs^22^. Because of the important metabolic impact of IGN, the changes we observed in hepatic metabolism of GF mice compared to SPF mice could result from the known impairment of IGN in GF mice. To address this question, we used mice with an intestinal-specific knockout of the catalytic subunit of glucose-6-phosphatase (I-G6pcKO), the essential enzyme of gluconeogenesis^23^. We tested whether the absence of IGN would modulate the expression of some hepatic genes, which exhibit modified expression in GF mice. To do so, we analysed the clock genes *Rev-Erbα*, *Bmal1*, and *Per2* (Fig. 5a), *Cyp4a14*, *Fgf21* (Fig. 5b), *Fasn*, *Lpk* (Fig. 5c), *Cyp2b10*, and *Cyp3a11* (Fig. 5d), whose daily expression is dramatically modified in GF mice (Fig. 3). The expression of most of these genes at ZT6 in I-G6pcKO SPF mice was similar to that of wild-type mice, with the expression of Cyp genes increased in IGN-deficient mice (Fig. 5). At ZT18, wild-type and mutant mice did not differ for any genes tested. These results suggest that the changes in hepatic metabolic oscillations induced by the lack of a gut microbiome are not caused by the reduction in IGN observed under GF conditions^12^.

### Daily changes in hepatic metabolites and plasma markers in GF mice

The results presented so far suggest a strong influence of the gut microbiome on liver metabolism. To test the outcome of the observed modifications in gene expression on liver physiology, we next performed an unbiased hepatic metabolic profiling by nuclear magnetic resonance (NMR) to assess whether the absence of a gut microbiome in GF mice influences the course of daily hepatic metabolite abundance. Modifications of the metabolite profiles at the four time points (ZT0/ZT6/ZT12/ZT18) would demonstrate that gene expression changes are translated into modifications in metabolic pathways activity.

**Figure 6:**
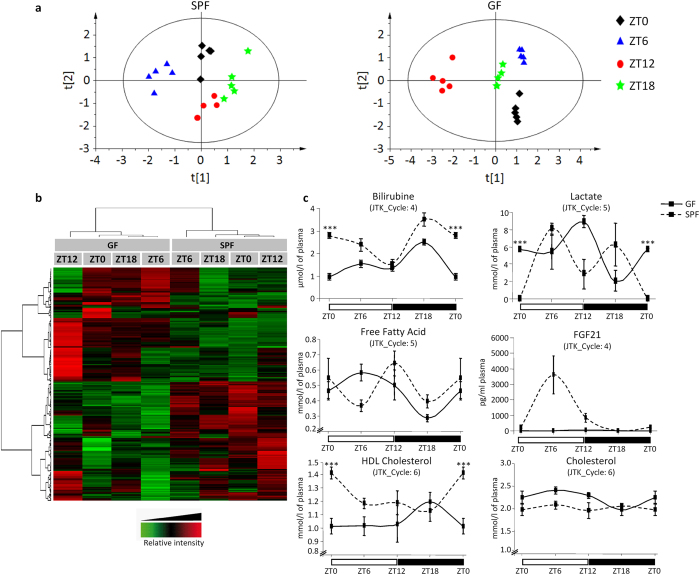
Gut microbiome influences hepatic metabolites and plasma biomarkers. (**a**) Two-dimensional PLS-DA scores plot of liver extract integrated ^1^H-NMR spectra. Each dot represents an observation (animal), projected onto first (horizontal axis) and second (vertical axis) PLS-DA variables. Time points are shown in different colors: ZT0 in black, ZT6 in blue, ZT12 in red and ZT18 in green. The black ellipse determines the 95% confidence interval, which is drawn using Hotelling’s T^2^ statistic. Left: SPF mice: A = 5, R^2^ = 94.6%, Q^2^ = 0.654; Right GF mice: A = 4, R^2^ = 87.5%, Q^2^ = 0.616. (**b**) Periodic changes in metabolites detected by NMR profiling of liver metabolites from GF and SPF mice are presented as a heatmap. Red and green indicate values above and below the mean, respectively. Black indicates values close to the mean. Individual values for each group are represented in the heatmap, and the hierarchical clustering was obtained from individual values using 1-Pearson correlation coefficient as distance and the Ward’s criterion for agglomeration. (**c**) Plasma biochemistry in GF (black line) and SPF (black dotted line) mice. Data are the mean ± SEM (n = 5 animals/group/time point) Statistical analyses were performed with Student’s t-test at each time point between SPF and GF mice. In case of unequal variances between the two samples, the Wehch’s two sample t-test was used. *P* values were corrected for multiple testing using BH procedure and FDR < 5% threshold is considered for significant difference. ***FDR < 0.005, **FDR < 0.01, *FDR < 0.05.

First, we used projection to latent structures for discriminant analysis (PLS-DA) to test whether there was a separation between groups of observations made in both GF and SPF mice at the four ZT time points (Fig. 6a). The SPF observations are clearly discriminated (five latent variable – PLS-DA model: R^2^ = 94.6% and Q^2^ = 0.654) according to the time points. A total of 114 buckets were selected as having a VIP value higher than 1.5 and as significantly different among the four groups (*P* < 0.05, Kruskal–Wallis test). A valid and robust PLS-DA model comprising four latent variables was also constructed on OSC-filtered and Pareto-scaled data from the GF mice. The four groups were also discriminated (R^2^ = 87.5% and Q^2^ = 0.616). A total of 120 buckets were selected as having a VIP value higher than 1.5 and as significantly different among the four groups (*P* < 0.05, Kruskal–Wallis test). These results indicated dynamic metabolite distributions according to sanitary status and sampling time points. Twenty-three representative metabolites with daily changes either in SPF or in GF mice are listed in Supplementary Table S4. Twenty of them showed a significantly different pattern between SPF and GF mice. Several metabolites, such as AMP, ADP, ATP, and NAD^+^, with different abundances in GF and SPF mice, are known modifiers of core clock gene activity in hepatocytes^24^,^25^. Furthermore, the metabolite changes also underscore an impact on protein metabolism (amino acids), glucose metabolism (lactate), nutrient metabolism (inosine), antioxidant defence (glutathione), and regulation of cellular processes (betaine).

Then, the average relative abundances of metabolites were used for hierarchical clustering as a heatmap (Fig. 6b). This heatmap revealed that (i) there is a distinct metabolic profile for each of the four ZT time points analysed (see also Fig. 6a); (ii) SPF and GF mice show markedly distinct daily metabolite patterns; and (iii) the metabolites cluster into two groups of similar size, with metabolites that are more abundant and metabolites that are less abundant in GF mice. These observations provided evidence for a major effect of the microbiome on the daily rhythmic oscillation in the hepatic metabolome, reflecting changes in metabolic activities. Similar to what was observed for gene expression, the absence of the microbiome did not abrogate oscillations but modified them profoundly.

Having observed that the absence of the microbiome heavily perturbed the expression correlation networks of PPARα and LXRβ, key factors in energy metabolism, we next addressed whether the daily oscillation of the abundance of plasma biochemical markers also was associated with alterations in metabolites in the liver. To test this hypothesis, we measured the daily concentrations of 6 markers in the plasma of GF and SPF mice (Fig. 6c). Among the markers tested, HDL cholesterol, bilirubine and lactate displayed significant changes at ZT0 in their concentrations in GF mice compared to SPF mice (see Supplementary Table S5 online). Furthermore, bilirubine, lactate, free fatty acid and FGF21 presented a daily pattern altered in GF compared to SPF mice (JTK_Cycle) (see Supplementary Table S6 online).

Collectively, these results demonstrated a severely modified hepatic metabolism in GF mice, with a systemic impact on circulating biomarkers.

## Discussion

The beneficial adaptation of organisms to the natural light–dark cycle is reflected in biological rhythms, which includes oscillations of diverse metabolic functions in peripheral organs adjusted to the feeding time^3^. Work by Schibler and colleagues^15^ showed that circadian gene expression in peripheral organs is connected to food intake, and food processing is a major purpose of circadian gene expression in mouse liver. In all animals, food intake is intimately associated with the gut microbiome, which increases energy extraction from nutrients.

The results presented here further underscore the existence of a gut microbiome–liver axis and provide novel evidence that the gut microbes not only participate in energy harvest but also control the rhythm and amplitude of liver daily oscillations. We found that the expression of the core clock genes was affected in the absence of the microbiome. Oscillation of these genes was not erased but was altered with modifications of either the nadir (*Bmal1*) or the zenith (*Per1, Cry1, Rev-erbβ*). In a recent similar study, reduced expression of *Bmal1* and *Per2* was observed in GF livers, which we did not see herein, but the same modifications in general canonical liver transcriptomic signatures were found in both the present report and the mentioned study^14^. Because the gut microbiome actively participates in gaining energy from food and in producing diet-derived products, the question of its impact on peripheral clock functions in metabolic organs is important^26^. It is already known that the gut microbiome controls the intestinal clock, as studied in the large intestine, where a large and diverse microbial population thrives^12^. Moreover, in mammals, the microbiome undergoes daily oscillations, which are altered in mice with a mutated clock system as well as during jet-lag (phase-reversal) experiments^13^. Interestingly, rhythmicity of the intestinal microbiota is more pronounced in females than in males^27^. In the present work performed with germ free animals, we show that male GF mice, chosen because their liver is not influenced by the estrous cycle, not only display a major defect in the expression profile of core clock genes but also in the expression of clock output effectors, such as the transcription factors Dbp, Dec2/Bhlhb42, and Tef. Consequently, the effects on liver metabolism are very broad, as indicated by our liver transcriptome analysis. Genes belonging to the response to superoxide, glutathione metabolic, and oxido-reduction processes are downregulated, showing a possible vulnerability of the GF mice to oxidative stress. Such alterations have been linked to a change in the cyclic activity of mitochondrial SIRT3, which is regulated by cycling levels of NAD^+^^28^. In contrast, increased expression of genes belonging to the activation of the immune response, proteolysis, the protein activation cascade, and the carboxylic acid catabolic process may reflect a response of the hepatic immune system to the lack of a microbiome and a stimulation of protein metabolism. Thus, the lack of a gut microbiome modifies the robust hepatic daily oscillations of the expression of genes involved in stress response and detoxification and also of several metabolic proteins.

However, at this stage, we cannot formally exclude that other mechanisms may participate in the phenotype observed, such as hormonal and/or humoral mechanisms, effects of other rhythmic processes in the body, or any other physiological factors that could be affected by the absence of microbiota, which in turn could impact daily rhythms in the liver and/or in the plasma.

Increased expression of genes of autophagic vacuole assembly, response to starvation, and negative regulation of cholesterol absorption at ZT6 may indicate an energy deficiency in GF mice during the resting light phase. As noted above, gut microbes largely contribute to maximizing energy gain from food; therefore, the GF mice may experience an energy crisis during the light and resting phases. Consistent with this possibility is the observation that the daily expression of PPARα target genes and the PPARα correlation network are impaired in GF mice when compared to SPF animals. Indeed, PPARα plays a critical role in autophagy^29^ as well as in glucose and lipid catabolism during fasting^30^; it is also a major player in whole body cholesterol homeostasis^31^. Although fasting-induced PPARα-dependent hepatic ketosis is impaired in GF mice^32^, our data provide evidence that the lack of a microbiome is sufficient to alter the constitutive daily activity of hepatic PPARα. However, there was no impact on light phase locomotor activity, which suggests no modification in the global light/dark eating pattern.

Of greatest interest, the daily oscillations of the nuclear receptors in the liver of GF mice did not appear to be profoundly modified. In liver, 20 nuclear receptors are expressed periodically^17^, and their regulation is often multifactorial, as for PPARα^33^,^34^. Most surprising, however, is the observation that the downstream target genes for PPARα, PXR, CAR, and LXRα were severely affected. IGN is critical for the beneficial metabolic effects of microbiome-derived molecules^22^, so we asked whether IGN participates in the control of the liver clock and the activity of these nuclear receptors whose daily activity is impaired in GF mice. Reduced IGN under SPF conditions did not significantly modify the expression of a set of genes involved in the liver clock or change the expression of a representative group of genes regulated by fatty acid (PPARs), oxysterol (LXRs), and xenobiotic (CAR, PXR) sensors, findings similar to those for GF mice. Thus, other molecular mechanisms such as receptor posttranslational modifications, co-regulator availability, or epigenetic changes and, most important, altered ligand production may have roles in governing liver clock functions. In fact, we previously showed that in liver, the PAR bZip transcription factors DBP, hepatic leukaemia factor (HLF), and TEF contribute to the daily transcription of acyl-CoA thioesterases, resulting in a cyclic release of fatty acids from thioesters. In turn, these fatty acids activate PPARα, which stimulates the expression of genes whose products are involved in the uptake and metabolism of lipids, cholesterol, and glucose^35^. Similarly, because PPARβ/δ and LXRβ cooperate in the regulation of bile acid homeostasis^36^, the disruption of their networks may be reflected in modifications in bile acid metabolism in GF mice, affecting their activity^37^.

Given that a majority of cellular functions are regulated at several levels, we studied the oscillations of hepatic metabolites as a clear output of the observed gene expression or other regulatory modifications. Metabolite abundance changes are expected to reflect modifications in the intricate metabolic activities from gene expression to protein activity. We found that metabolites oscillated in both GF and SPF mice but that their rhythmic abundances largely differed between these mice. Recent work with a comprehensive dataset of over 500 metabolites highlighted the coordinate clock-controlled oscillation of many metabolites comprising the amino acid, carbohydrate, lipid, nucleotide, and xenobiotic metabolic pathways^38^. We found that this coordination was severely altered in the GF mice, which was anticipated from the disrupted regulation of liver clock gene function.

The importance of the regulatory role of the gut microbiome was also revealed by the alteration, in its absence, of the abundance and oscillation of several plasma markers associated with liver functions, particularly energy homeostasis. Compared to the levels observed in mice exposed to bacteria, some of these markers showed an advanced phase shift towards the light phase (free fatty acids). Furthermore, the levels of HDL cholesterol were increased and those of bilirubin decreased. In mouse plasma, HDL represents 75–80% of the lipoprotein fraction, and its robust increase in GF mice suggests a perturbation in cholesterol metabolism. In addition, higher cholesterol levels correlated with low bilirubin levels. In humans, low bilirubin is linked to diseases associated with oxidative stress^39^ and to an increased risk for cardiovascular disease and stroke^40^. Low serum total bilirubin levels are also found in familial hypercholesterolemia patients with cardiovascular disease^41^. Furthermore, it has been proposed that bilirubin, haemoglobin, and blood-borne melatonin could act in concert to shift the phase of the clock^42^. Finally, the daily expression of FGF21 was completely abolished in the GF mice, which is explained at least in part by the arrested daily PPARα activity.

In conclusion, our work demonstrates that the gut microbiome appears to be very important in establishing physiological oscillations in the liver and further supports a recent report showing that bacterial metabolites can influence daily clock gene expression within hepatocytes^14^. Our data show that the gut microbiome regulates core clock genes, their effectors, and the activity of key nuclear receptors, resulting in broad changes in rhythmic gene expression and hepatic metabolites. Therefore, specifically assembled food taken in a timely manner could affect liver function; indeed, food intake in mice and humans follows a daily cycle, and alterations in microbiome composition and diversity are frequently observed following diet changes^43^,^44^. The liver responds to changes in the gut microbiome most likely via cues carried through the portal vein, which can serve as an entry for bacteria and bacteria-generated metabolites. In fact, the gut microbiome has already been reported to be associated with liver function in health and disease^2^,^14^,^45^,^46^. To fully understand the pathophysiology of several liver diseases, including non-alcoholic fatty liver disease, non-alcoholic steatohepatitis, and hepatocarcinogenesis^47^,^48^, it is important to consider the implications of the daily oscillations of liver functions. Altogether, this work emphasizes that the gut microbiome, which is required for optimal energy harvest from nutrients, is also involved in collecting or producing signals that affect the daily activity of the liver.

## Methods

### Animals

Mice were housed at the Core Facility for Germfree Research (CFGR), Karolinska Institute Stockholm, or at the Germfree Facility of Lee Kong Chian School of Medicine, Nanyang Technological University, Singapore under either strict axenic (germ-free, GF) or conventional specific pathogen–free conditions (SPF). All mice were maintained on autoclaved R36 Lactamin (Stockholm, Sweden) or autoclavable Labdiet 5010 (Singapore) chow on a 12-hour light (ZT0-ZT12) 12-hour dark (ZT12-ZT24) cycle. ZT stands for Zeitgeber time; ZT0 is defined as the time when the lights are turned on and ZT12 as the time when lights are turned off. GF mice were bred and maintained in germ-free plastic isolators. SPF mice used in experiments were born from SPF mothers placed before delivery in the same plastic isolators as those used for maintenance of germ-free mice. All food, bedding material, and water were similar for germ-free and SPF mice. Protocols involving the use of GF and SPF animals were approved by the Regional Animal Research Ethical Board, Stockholm, Sweden (Stockholms norra djurförsöksetiska nämnd), and followed proceedings described in the EU legislation (Council Directive 86/609/EEC). Animal husbandry was in accordance with Karolinska Institute guidelines and approved by the above-mentioned ethical board. For the animals handled in Singapore, the protocols were approved by the Institutional Animal Care and Use Committee (2012/SHS/743).

Ten to twelve week-old C57Bl/6 male mice were killed by cervical dislocation at four time points in a pair-wise manner: ZT0, ZT6, ZT12 and ZT18. Blood was collected prior to sacrifice at the submandibular vein with a lancet in EDTA-coated tubes. Plasma were prepared by centrifugation (1500 g, 10 min, 4 °C) and kept at −80 °C. Liver was removed, snap-frozen in liquid nitrogen and stored at −80 °C until use.

Twenty-four hour open field test. To measure general activity, mice were individually placed into a Plexiglas cage (40.5 cm × 40.5 cm × 16 cm) for 24-hour open-field testing. Behavioural measures such as horizontal activity and mobility time were monitored using the Versamax program (AccuScan Instruments Inc., Columbus, OH, USA). Statistics were performed with Student’s t-test. In case of unequal variances between the two samples, the Wehch’s two sample t-test was used. FDR < 5% threshold is considered for significant difference.

Intestine-specific G6pc-null mice (I-G6PcKO) were previously described^49^ and were housed in the SPF animal facility of Lyon 1 University under controlled temperature conditions with a 12-hour light 12-hour dark cycle. The mice had free access to water and to a standard chow diet (Safe 04). 15 week-old I-G6Pc and C57Bl/6J (WT) male mice were sacrificed by cervical dislocation at ZT6 and ZT16 (n = 6 animals/genotype/time point). Livers were collected and snap frozen in liquid nitrogen and kept at −80 °C for further used. All procedures concerning these mice were performed in accordance with the EU legislation (Council Directive 86/609/EEC). The regional animal care committee (CREEA CNRS, Rhône Alpes, Auvergne, France) approved all the experiments.

### Gene expression studies

Total RNA was extracted with TRIzol reagent (Invitrogen). RNA amounts were determined using the NanoDrop®ND-1000, and the RNA quality was assessed using RNA 6000 NanoChips with the Agilent 2100 Bioanalyzer (Agilent, Palo Alto, CA, USA). For each sample, 100 ng of total RNA was amplified using the WT Sense Strand Target Labelling kit (Affymetrix, Cat. no. 900223); 5.5 μg of the resulting sense cDNA was fragmented by UDG (uracil DNA glycosylase) and APE 1 (apurinic/apyrimidic endonuclease 1) and biotin-labelled with TdT (terminal deoxynucleotidyl transferase) using the GeneChip® WT Terminal Labelling kit (Affymetrix Cat. no. 900671, Santa Clara, CA, USA). Affymetrix Mouse Gene 2.0 ST arrays (Affymetrix, Santa Clara, CA, USA) were hybridized with 2.7 μg of biotinylated target at 45 °C for 17 hours and washed and stained according to the protocol described in the Affymetrix GeneChip® Expression Analysis Manual (Fluidics protocol FS450_0007). The arrays were scanned with a GeneChip® Scanner 3000 7G (Affymetrix). Normalized expression signals were calculated from Affymetrix CEL files by the Robust Multi-array Average algorithm, using the Affymetrix Expression Console Software (version 1.3.0.187). Hybridization quality also was assessed with Expression Console Software. All data were analysed using R (www.r-project.org). Microarray data analysis was conducted using Bioconductor packages (www.bioconductor.org, v 2.12,^50^ as described in GEO entry GSE71628). A model was fitted using the limma lmFit function^51^. A correction for multiple testing was then applied using Benjamini-Hochberg (BH) procedure for the false discovery rate (FDR)^52^. ProbeSets with (BH) adjusted *P* values ≤ 0.01 were considered to be differentially expressed between conditions. Hierarchical clustering was applied to the samples and the differentially expressed probeSets using 1-Pearson correlation coefficient as distance and Ward’s criterion for agglomeration. The enrichment of Gene Ontology (GO) Biological Processes was evaluated using a conditional hypergeometric test (GOstats package)^53^.

Networks of the 50 genes having the highest absolute correlation (Pearson-correlation) with a transcription factor of interest (red node) in SPF mice were displayed using the R function circlePlot^54^. Only the edges corresponding to significant correlations were represented (Bonferroni-adjusted *P* value < 5%). Positive and negative correlations were represented by red and blue edges, respectively.

Correlation matrices between genes of interest in SPF and GF conditions were also represented as color-coded tables using the *labeledHeatmap* function of the WGCNA R package^55^. In this representation, each cell contains the correlation between two genes and the corresponding p-value. The tables are color-coded by correlations according to the color legend (red for positive correlations and green for negative correlations). We colored in grey the cells corresponding to non-significant correlations (p-value threshold of 5%).

For real-time quantitative polymerase chain reaction (qPCR), total RNA samples (2 μg) were reverse-transcribed using the High Capacity cDNA Reverse Transcription Kit (Applied Biosystems). Primers for SYBR Green assays are listed in Supplementary Table S7. Amplifications were performed on an ABI Prism 7300 Real Time PCR System (Applied Biosystems). qPCR data were normalized by TATA-box binding protein mRNA levels and analysed with LinRegPCRv2012.2^56^. Statistical analyses for qPCR data were performed with Student’s t-test at each time point between SPF and GF mice. In case of unequal variances between the two groups, the Wehch’s two sample t-test was used. *P* values were corrected for multiple testing using BH procedure and FDR < 5% threshold is considered as significant differences.

### Metabolomic analyses by ^1^H nuclear magnetic resonance (NMR) spectroscopy

See Supplementary Methods online.

### Multivariate analysis of metabolomic data

The pre-processed NMR data were imported into Simca-P^+^ software (version 13.0.2, Umetrics AB, Umea, Sweden) for multivariate statistical analysis. First, principal component analysis was used to detect intrinsic clusters and outliers. Then, partial least squares–discriminant analysis (PLS–DA) was used to study the relationship between time and NMR spectral data. Seven-fold cross validation was used to determine the number of latent variables to include in PLS-DA models. R^2^ (proportion of explained variation) and Q^2^ (predictive ability) values were used to assess PLS-DA model validity. Typically, a valid model has R^2^ > 50% and Q^2^ > 0.4. A permutation test (200 iterations) was used to assess PLS-DA model robustness. Discriminant NMR buckets were selected using the very important variable (VIP) value, a global measure of the influence of each variable on the PLS-DA model. The non-parametric Kruskal–Wallis test was finally used to determine significant variables among the VIP-selected variables. This test was conducted using R software.

NMR data were preprocessed using orthogonal signal correction (OSC) with time as a correction factor. The OSC filtering was applied to remove variation in the NMR matrix data not correlated with the time (confounding factors such as physiological, experimental, or instrumental variation). Filtered data were then Pareto scaled. PLS-DA was applied to the filtered and scaled data. Hierarchical clustering and a heatmap were used to classify averaged NMR data according to phenotypes (SPF vs GF) over Zeitgeber time (ZT0/ZT6/ZT12/ZT18). In this method, identical NMR profiles are clustered together. The heatmap was drawn using the R “gplots” package (http://www.r-project.org/).

### Plasma biochemistry

See Supplementary Methods online.

### Rhythmicity analysis for liver gene expression, liver metabolomic and plasma biochemistry data

For each data set, statistical tests for rhythmicity were performed using JTK_Cycle algorithm^16^ (http://dx.doi.org/10.1177/0748730410379711), reporting BH adjusted *P* values for the identification of waveform parameters for each variable (ProbeSet for microarray, gene for qPCR, bucket for metabolomic, biochemical compound), and BH adjusted *P* values for multiple testing between all variables. Variables with FDR < 5% were considered significantly rhythmic.

Variables were then classified into 6 different classes according their rhythmicity significance and parameters (period and phase lag) regarding SPF and GF mice. Class 1: significant rhythmic variables and identical parameters results for both SPF and GF mice; Class 2: significant rhythmic variables in both SPF and GF mice but with a phase lag in GF mice compared to SPF; Class 3: significant rhythmic variables in both SPF and GF mice but with a different period in GF mice compared to SPF; Class 4: significant rhythmic variables in SPF but not in GF mice; Class 5: significant rhythmic variables in GF but not in SPF mice; Class 6: no significant rhythmic variables neither in SPF nor in GF mice.

## Additional Information

**How to cite this article**: Montagner, A. *et al.* Hepatic circadian clock oscillators and nuclear receptors integrate microbiome-derived signals. *Sci. Rep.* **6**, 20127; doi: 10.1038/srep20127 (2016).

## Supplementary Material

Supplementary Information

Supplementary Excel File
