# Erratum: Hepatic circadian clock oscillators and nuclear receptors integrate microbiome-derived signals

**DOI:** 10.1038/srep23951

**Published:** 2016-04-20

**Authors:** Alexandra Montagner, Agata Korecka, Arnaud Polizzi, Yannick Lippi, Yuna Blum, Cécile Canlet, Marie Tremblay-Franco, Amandine Gautier-Stein, Rémy Burcelin, Yi-Chun Yen, Hyunsoo Shawn Je, Maha Al-Asmakh, Gilles Mithieux, Velmurugesan Arulampalam, Sandrine Lagarrigue, Hervé Guillou, Sven Pettersson, Walter Wahli

Scientific Reports 6: Article number: 2012710.1038/srep20127; published online: 02162016; updated: 04202016.

The original version of this Article contained an error in the spelling of the author Maha Al-Asmakh, which was incorrectly given as Al-Asmakh Maha. This has now been corrected in the PDF and HTML versions of the Article.

