# Supplementary material for: Hepatic circadian clock oscillators and nuclear receptors integrate microbiome-derived signals
Source: Sci Rep. 2016 Feb 16;6:20127. doi: 10.1038/srep20127 (PMC4754633; doi:10.1038/srep20127)
Supplement: Supplementary Information [file srep20127-s1.pdf]

Hepatic circadian clock oscillators and nuclear receptors integrate microbiome-derived signals

Alexandra Montagner[1], Agata Korecka[2], Arnaud Polizzi[1], Yannick Lippi[1], Yuna Blum[3], Cécile Canlet[1], Marie Tremblay-Franco[1], Amandine Gautier-Stein[4], Rémy Burcelin[5], Yi-Chun Yen[6], Hyunsoo Shawn Je[6], Maha Al-Asmakh[2,7], Gilles Mithieux[4], Velmurugesan Arulampalam[2], Sandrine Lagarrigue[8], Hervé Guillou[1*], Sven Pettersson[2,9*], Walter Wahli[1,9,10*]

[1] INRA ToxAlim, UMR1331, Chemin de Tournefeuille, Toulouse Cedex, France

[2]Department of Microbiology, Tumor and Cell Biology (MTC), Karolinska Institutet, Stockholm, Sweden

[3]Department of Medicine/Division of Cardiology, UCLA, Los Angeles, USA

[4]Institut National de la Santé et de la Recherche Médicale, U855, Lyon, France

[5]Institut des Maladies Métaboliques et Cardiovasculaires, Hôpital Rangueil, Toulouse Cedex, France

[6]Molecular Neurophysiology Laboratory, Signature Program in Neuroscience and Behavioral Disorders, Duke-NUS Graduate Medical School, Singapore

Department of Physiology, Yong Loo Lin School of Medicine, National University of Singapore, Singapore

[7]Department of Health Sciences, College of Arts and Sciences, Qatar University, Daha, Qatar

[8]INRA, UMR1348 Pegase, Saint-Gilles; Agrocampus Ouest, UMR1348 Pegase, Rennes; Université Européenne de Bretagne, France

[9]Lee Kong Chian School of Medicine, Nanyang Technological University, Singapore

[10]Center for Integrative Genomics, University of Lausanne, Le Genopode, Lausanne, Switzerland

***Corresponding authors:**

Walter Wahli, Lee Kong Chian School of Medicine, Nanyang Technological University, Singapore, The Academia, 20 College Road, Singapore 169856
E-mail: walter.wahli@ntu.edu.sg

Sven Pettersson, Lee Kong Chian School of Medicine, Nanyang Technological University, Singapore, The Academia, 20 College Road, Singapore 169856, Karolinska Institutet, MTC, Nobels way 16 17177 Solna, Sweden
E-mail: sven.pettersson@ki.se

Hervé Guillou, INRA ToxAlim, UMR1331, Chemin de Tournefeuille, Toulouse Cedex, France
E-mail: herve.guillou@toulouse.inra.fr

Additional information

Additional Methods

Metabolomic analyses by ^{1}H nuclear magnetic resonance (NMR) spectroscopy. ^{1}H NMR spectroscopy was performed on aqueous liver extracts prepared from liver samples (50–75 mg) homogenized in chloroform/methanol/NaCl 0.9% (2/1/0.6) containing 0.1% butyl hydroxytoluene and centrifuged at 5000 ×*g* for 10 min. The supernatant was collected, lyophilized, and reconstituted in 600 μL of D_2O containing 0.25 mM TSP [3-(trimethylsilyl)propionic-(2,2,3,3-d4) acid sodium salt] as a chemical shift reference at 0 ppm. All ^{1}H NMR spectra were obtained on a Bruker DRX-600 Avance NMR spectrometer operating at 600.13 MHz for ^{1}H resonance frequency using an inverse detection 5 mm ^{1}H-^{13}C-^{15}N cryoprobe attached to a CryoPlatform (the preamplifier cooling unit). The ^{1}H NMR spectra were acquired at 300 K with a 1D NOESY-presat sequence (relaxation delay – 90°-t-90°-tm-90°-acquisition). A total of 128 transients were acquired into a spectrum with 20 ppm width, 32 k data points, a relaxation delay of 2.0 s, and a mixing delay of 100 ms. All ^{1}H spectra were zero-filled to 64 k points and subjected to 0.3 Hz exponential line broadening before Fourier transformation. The spectra were phase and baseline corrected and referenced to TSP (^{1}H, d 0.0 ppm) using Bruker Topspin 2.1 software (Bruker GmbH, Karlsruhe, Germany).

To confirm the chemical structure of the metabolites of interest, 2D ^{1}H-^{1}H COSY (correlation spectroscopy) and 2D ^{1}H-^{13}C-HSQC (heteronuclear single quantum coherence spectroscopy) NMR experiments were performed on selected samples. Spectral assignment was based on matching 1D and 2D data to reference spectra in a lab-built reference database, as well as with other databases (http://www.brmb.wisc.edu and http://www.hmdb.ca/) and reports in the literature.

All NMR spectra were reduced using AMIX software (version 3.9, Bruker, Analytik) to integrate 0.01 ppm–wide regions corresponding to the d 9.4–0.8 ppm region. The 5.1–4.4 ppm region, which includes the water resonance, was excluded. A total of 788 NMR buckets were included in the data matrices. To account for differences in sample amount, each integrated region was normalized to the total spectral area.

Plasma biochemistry. Plasma levels of total cholesterol, high-density lipoprotein (HDL) cholesterol, free fatty acids, bilirubin and lactate were determined on a biochemical analyser, COBAS – MIRA +. Plasma level of FGF21 was determined using the Rat/Mouse FGF21 Elisa Kit (Merck Millipore, EZRMF FGF21-26K) according to the manufacturer's instructions. Statistical analyses for biochemistry data were performed with Student's test at each time point between SPF and GF mice. In case of unequal variances between the two samples, the Wehch's two sample t-test was used. *P* values were corrected for multiple testing using BH procedure and FDR<5% threshold is considered for significant differences.

Additional figure legends

Supplementary Table S1. Results of rhythmicity analysis (JTK_Cycle) on qPCR data. The results of JTK_Cycle analysis of the qPCR gene expression data are presented. Each line corresponds to a tested gene (Gene ID) and shows result from JTK_Cycle analysis: BH adjusted *P* values (BH.pvalue) for gene multiplicity testing; JTK_Cycle best BH adjusted *P* value for waveform parameters testing (JTK.ADJP); period (PERIOD), phase lag (LAG) and amplitude (AMP) for SPF and GF, respectively. Last column contains rhythmicity class determined as described in the legend of Figure 2c.

Supplementary Table S2. Statistical analysis on qPCR data. Statistical analyses for qPCR data were performed with Student's t-test at each time point between SPF and GF mice. In case of unequal variances between the two samples, the Wehch's two sample t-test was used. *P* values were corrected for multiple testing using BH procedure and FDR<5% threshold is considered for significant difference. The first 4 columns correspond to variance equality test results (1 for equality; 0 for inequality). The last 4 columns corresponds to BH adjusted *P* values.

Supplementary Figure S1. Color-coded gene correlations matrix between core clock gene regulators and effectors in liver of GF and SPF mice. The matrices are symmetric and the diagonal contains the correlations between each gene and itself. Each cell contains the correlation between two genes and the corresponding *P* value. The tables are color-coded by correlation according to the color legend (red and green for positive and negative correlations, respectively). Grey cells correspond to non-significant correlations (*P* value threshold of 5%).

Supplementary Figure S2. Locomotor activity of GF versus SPF mice. Male mice were individually placed into a Plexiglas cage for 24-hour open-field testing (n=4 animals/sanitary status). Behavioural measures such as horizontal activity and mobility time were monitored using the Versamax program. The average distance was calculated during the first light period (adaptation), dark period and next light period. Statistics were performed with Student's t-test. In case of unequal variances between the two samples, the Wehch's two sample t-test was used. FDR<5% threshold is considered for significant difference. * FDR< 0.05.

Supplementary Table S3. Major enriched biological processes impacted by gut microbiome. The most significantly enriched GO biological functions (*P* value < 0.001; GOstats R package analysis) are presented for each probeSet cluster presented in Figure 2a. ProbeSet cluster number, number of differentially expressed probeSets in liver samples of GF vs SPF and associated top significant GO functions are illustrated.

Supplementary Figure S3. Gut microbiome effects on hepatic gene expression correlation networks of *Cry1, Rev-erbα, Rorγ and Per2*. Networks of the 50 genes showing the highest absolute correlation with *Cry1*, *Rev-erbα*, *Rorγ*, and *Per2* under SPF conditions are presented as circle plots. The edges corresponding to significant correlations are represented (Bonferroni-adjusted *P* value < 5%). The thickness of the edges reflects the absolute correlation, and red/blue were used for positive/negative correlations, respectively. The size of each node indicates the connectivity in the circle plots. Magenta nodes correspond to genes significantly correlated with the gene of interest (adjusted P value < 5%). The correlation within the same network is then presented in GF mice.

Supplementary Figure S4. Gut microbiome effects on hepatic gene expression correlation networks of *Clock, Bmal1, Rev-erbβ, Per1, Cry2, and Rorα*. Networks of the 50 genes showing the highest absolute correlation with *Clock, Bmal1, Rev-erbβ, Per1, Cry2, and Rorα* expression under SPF conditions are presented as circle plots. Networks of the 50 genes having the highest absolute correlation with the transcription factor of interest (red node) in SPF mice are displayed. The edges corresponding to significant correlations are represented (Bonferroni-adjusted P value < 5%). See Legend of Supplementary Fig. S3 for further explanation.

Supplementary Figure S5. Gut microbiome effects on hepatic gene expression correlation networks of *Pparα* at each ZT time point. Networks of the 50 genes showing the highest absolute correlation with *Pparα* expression under SPF condition at ZT0, ZT6, ZT12, and ZT18 (n= 5 mice/ZT time point) are presented as circle plots. The edges corresponding to significant correlations are only represented (Bonferroni-adjusted P value < 5%). Another network circle plot based on these 51 genes is then presented in GF mice. The edges corresponding to significant correlations are represented (Bonferroni-adjusted P value < 5%). See Legend of Supplementary Fig. S3 for further explanation.

Supplementary Figure S6. Gut microbiome effects on hepatic gene expression correlation networks of *Pparβ/δ* at each ZT time point. Networks of the 50 genes showing the highest absolute correlation with *Pparβ/δ* expression under SPF condition at ZT0, ZT6, ZT12, and ZT18 (n= 5 mice/ZT time point) are presented as circle plots. The edges corresponding to significant correlations are only represented (Bonferroni-adjusted P value < 5%). Another network circle plot based on these 51 genes is then presented in GF mice. The edges corresponding to significant

correlations are represented (Bonferroni-adjusted P value < 5%). See Legend of Supplementary Fig. S3 for further explanation.

Supplementary Table S4: Significant liver metabolites identified using PLS-DA modeling in GF and SPF mice and analysis of their rhythmic behavior using JTK_Cycle analysis. [1]*P* value of the Kruskal-Wallis test. PLS-DA modeling was used to identified discriminant metabolites (VIP > 1.5): GF: A= 5; R^2= 0.946; Q^2= 0.654; SPF: A= 5; R^2= 0.875; Q^2= 0.616. ZTn / ZTn+6 tests the relative difference in the abundance of a metabolite between n and n+6 hours in the day. A sign "+" indicates a significant increase in the relative abundance at n+6 compared to n. A sign "-" indicates a significant decrease in the relative abundance at n+6 compared to n. + or – in blue indicates a significant change that only occurs either in GF or in SPF mice [2]Rhythmic metabolites (Benjamini-Hochberg corrected *P* value < 0.05) identified using JTK_Cycle analysis. Class 1: significant rhythmic variable and identical parameters results for both SPF and GF mice; Class 4: significant rhythmic variable in SPF but not in GF mice; Class 5: significant rhythmic variable in GF but not in SPF mice.

Supplementary Table S5. Results of statistical analysis on biochemistry data. Statistical analyses were performed with Student's t-test at each time point between SPF and GF mice. In case of unequal variances between the two samples, the Wehch's two sample t-test was used. *P* values were corrected for multiple testing using BH procedure and FDR<5% threshold is considered for significant difference. The first 4 columns correspond to variance equality test results (1 for equality; 0 for inequality). The last 4 columns corresponds to BH adjusted *P* values.

Supplementary Table S6. Results of rhythmicity analysis of biochemistry data. The results of JTK_Cycle analysis of the biochemistry data are presented. Each line corresponds to a tested plasma marker (Marker ID) and shows result from JTK_Cycle analysis: BH adjusted *P* value (BH.pvalue) for marker multiplicity testing, JTK_Cycle best BH adjusted pvalue for waveform parameters testing (JTK.ADJP), period (PERIOD), phase lag (LAG) and amplitude (AMP) for SPF and GF, respectively. Last column contains rhythmicity class determined as described in the legend of Fig. 2c.

Supplementary Table S7. Sequence of primers used. Sequences of primer pairs, mRNA reference in NCBI of primers used in qPCR assays are presented.

Supplementary Excel File S1. Results of rhythmicity analysis (JTK_Cycle) on microarray data. The results of the JTK_Cycle analysis of gene expression microarray data are presented. Each line corresponds to a unique ProbeSet. Columns B, C and D refer to probeSet annotation; columns E to I contain JTK_Cycle analysis results for SPF mice: BH adjusted *P* values (BH.pvalue) for probeset multiplicity testing, JTK best BH adjusted pvalue for waveform parameters testing (JTK.ADJP), period (PERIOD), phase lag (LAG) and amplitude (AMP), respectively. Columns J to N contain JTK_Cycle analysis results for GF mice. Column O contains rhythmicity classes determined in defined in legend of Figure 2c. Column P contains the name of genes clusters identified in Figure 2a.

Gene ID	**SPF_BH.pvalue**	SPF_JTK.ADJP	SPF_PERIOD	SPF_LAG	SPF_AMP	**GF_BH.pvalue**	GF_JTK.ADJP	GF_PERIOD	GF_LAG	GF_AMP	Class
Bmal1	**0.000000564**	0.0000000537	24	0	1.243555272	**0.0000336**	0.000008	18	0	1.918461898	**3**
Reverba	**0.0000102**	0.00000243	18	9	4.426036965	**0.00000277**	0.000000132	24	9	1.885655157	**3**
Reverbb	**0.00000441**	0.000000631	24	12	2.250153222	**0.0000235**	0.00000448	18	9	2.121527548	**3**
Per1	**0.001739141**	0.000745346	24	12	1.364153761	**0.002278713**	0.000759571	18	9	4.69882926	**3**
Per2	**0.003190199**	0.001519142	24	15	26.97024045	**0.055511165**	0.029077277	24	15	19.00154627	**4**
Cry1	**0.0000102**	0.00000243	18	0	3.641490008	**0.001213699**	0.000346771	24	21	4.293000732	**3**
Dbp	**0.000000129**	0.00000000614	24	12	36.83328768	**0.00000313**	0.000000298	18	9	83.10661268	**3**
Dec2	**0.000257317**	0.000098	24	12	1.440305364	**0.000017**	0.00000243	18	9	3.597082161	**3**
Tef	**0.000217318**	0.0000621	24	12	4.709950848	**0.003544665**	0.001519142	18	9	8.674949904	**3**
Pxr	0.495681629	0.448473855	24	15	0.367085505	0.095887717	0.068491226	24	15	0.564856837	6
Cyp3a11	0.083732427	0.055821618	24	9	61.33892985	0.428690693	0.387863008	24	18	5.57407469	6
Car	0.639294741	0.608852135	18	12	0.742664272	0.78997342	0.752355638	24	21	0.997045065	6
Cyp2b10	**0.014115113**	0.00739363	18	3	1.202857261	**0.002810762**	0.001070766	24	18	0.986094365	**3**
Lxra	0.137912232	0.111643236	24	18	0.440111955	0.095887717	0.068491226	24	15	0.283747495	6
Fasn	0.063670028	0.036382873	24	18	9.722560343	0.813883849	0.813883849	12	6	1.920303527	6
PPARa	0.083732427	0.051917995	24	15	2.746145619	0.265783768	0.202501918	24	12	2.473828882	6
Fgf21	**0.000257317**	0.000098	24	6	0.408071899	0.095887717	0.058154554	12	3	0.064097223	**4**
Cyp4a14	**0.42616338**	0.365282897	12	0	17.81249969	**0.017872981**	0.008510943	18	15	6.125015325	**5**
Chrebpa	0.649947173	0.649947173	24	0	4.949150688	0.095887717	0.068491226	24	0	21.01773907	6
Chrebpb	0.113896481	0.081354629	24	12	1.657472221	0.307162295	0.248655191	24	6	0.862199387	6
Lpk	0.137912232	0.111643236	24	18	9.584292884	0.344931236	0.295655345	24	12	4.312581618	6

Gene ID	ZT0_var.equal	ZT6_var.equal	ZT12_var.equal	ZT18_var.equal	**ZT0_BH.adj.p**	**ZT6_BH.adj.p**	**ZT12_BH.adj.p**	**ZT18_BH.adj.p**
Bmal1	1	1	1	1	0.4550	**0.0184**	0.0549	**0.0031**
Reverba	1	1	1	0	0.2589	0.4196	**0.0045**	0.0862
Reverbb	1	1	0	1	0.1327	0.0850	0.0524	0.3986
Per1	1	0	1	1	0.6801	0.2027	0.0549	**0.0084**
Per2	0	0	1	1	0.3421	0.1050	0.7700	0.8860
Cry1	1	0	0	1	0.6219	0.4753	0.2358	**0.0149**
Dbp	1	0	1	1	0.2345	0.1050	0.0524	0.1637
Dec2	1	1	1	1	0.4884	**0.0079**	0.197085	0.4891
Tef	1	0	0	0	0.4884	0.2354	0.7128	0.3808
Pxr	1	1	1	1	0.2345	0.4753	0.7277	0.1637
Cyp3a11	0	0	0	1	**0.0070**	**0.0179**	**0.0031**	**5.18E-08**
Car	1	1	1	0	0.2345	0.9690	0.7128	0.0707
Cyp2b10	1	0	1	0	**0.0027**	0.0526	0.5484	**0.0149**
Lxra	1	0	0	1	0.2345	0.1887	0.1785	0.5362
Fasn	1	1	1	0	0.2137	0.1050	**0.0197**	**0.0105**
PPARa	1	1	1	1	0.0647	0.6124	0.6335	0.7623
Fgf21	0	0	0	1	0.0697	0.2627	0.9991	0.0627
Cyp4a14	0	0	1	0	0.0568	0.1050	**0.0045**	**0.0076**
Chrebpa	1	1	1	1	0.6219	0.7343	0.2358	0.1637
Chrebpb	1	1	1	0	0.1015	**0.0210**	**0.0045**	0.0714
Lpk	1	1	1	1	0.2589	0.7659	0.2358	**0.0257**

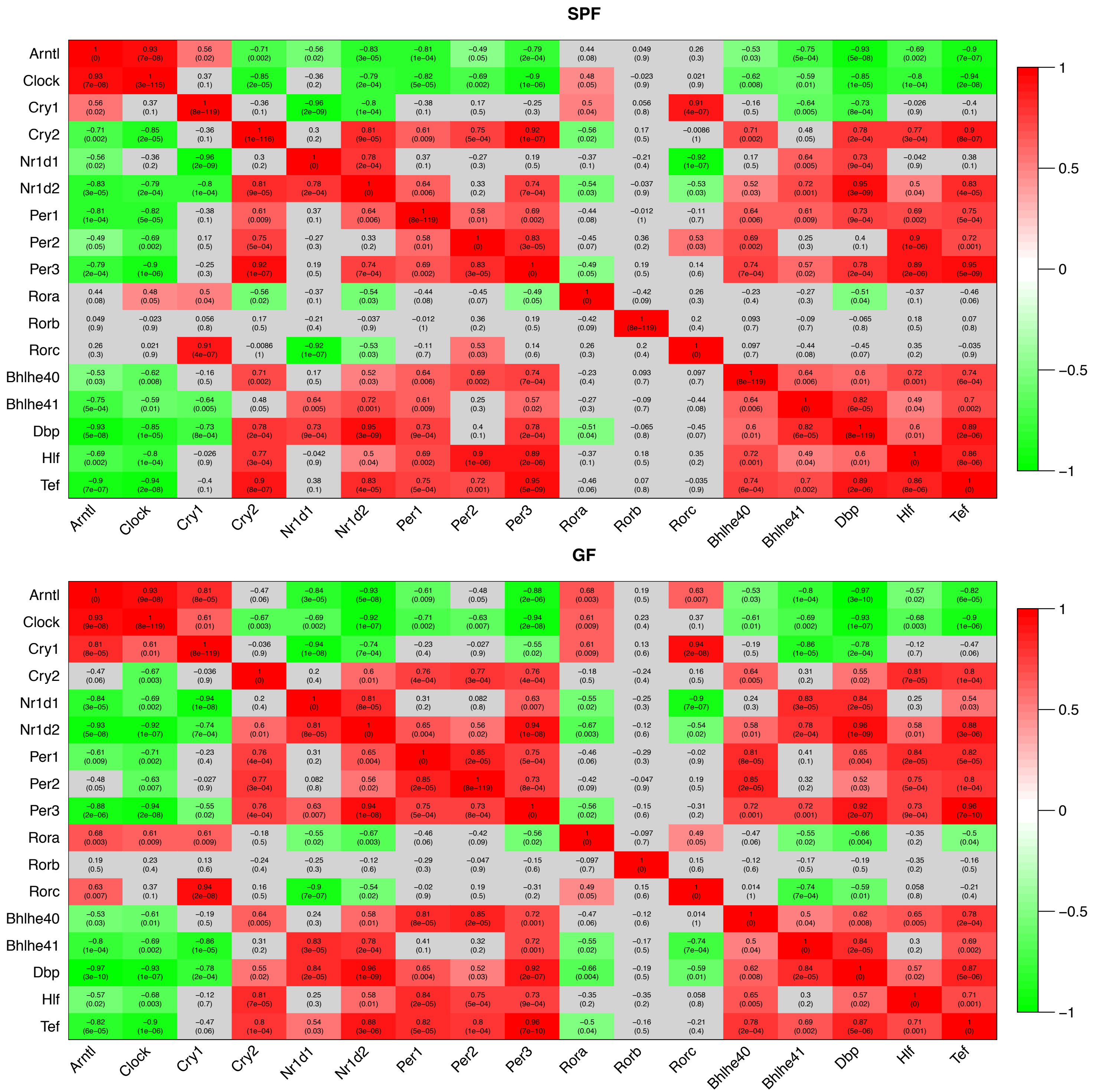
SPF
GF
Arntl
Clock
Cry1
Cry2
Nr1d1
Nr1d2
Per1
Per2
Per3
Rora
Rorb
Rorc
Bhlhe40
Bhlhe41
Dbp
Hlf
Tef
1
0.5
0
−0.5
−1

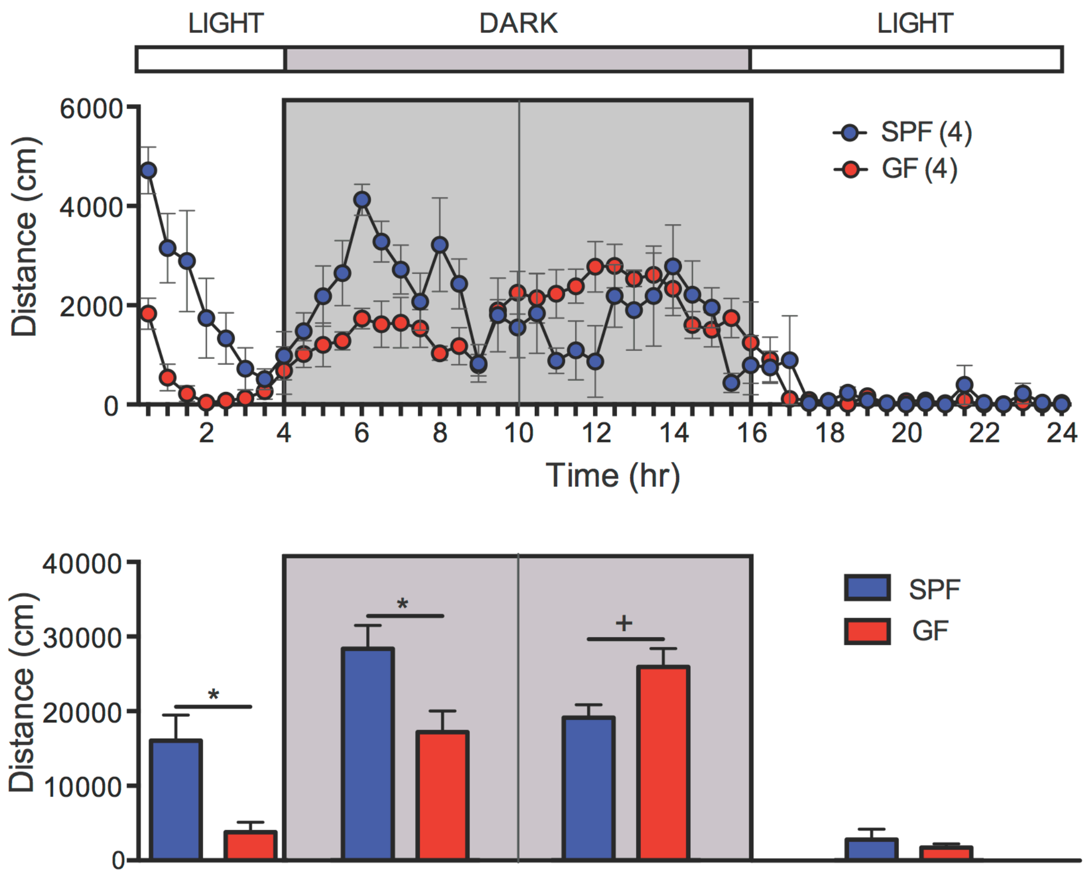

LIGHT
DARK
LIGHT
SPF (4)
GF (4)
Distance (cm)
Time (hr)
SPF
GF
Distance (cm)

Cluster nber	Nber of DEG in GF vs SPF	Top significant GO functions
9	616	GO:0006397 mRNA processing<br>GO:0008380 RNA splicing<br>GO:0051252 regulation of RNA metabolic process<br>GO:0006355 regulation of transcription, DNA-dependent<br>GO:0031326 regulation of cellular biosynthetic process<br>GO:0016568 chromatin modification
8	463	GO:0023052 Signaling<br>GO:0045579; GO:0045588 Positive regulation of B and g-d cell differentiation<br>GO:0042176 Regulation of protein catabolic process<br>GO:0032885 Regulation of polysaccharide biosynthetic process<br>GO:0014068 Positive regulation of phosphatidylinositol 3-kinase cascade
7	691	GO:0006749 Glutathione metabolic process<br>GO:0006082 Organic acid metabolic process<br>GO:0043603 Cellular amide metabolic process<br>GO:0055114 Oxidation-reduction process
6	423	GO:0006629 Lipid metabolic process<br>GO:0055114 Oxidation-reduction process<br>GO:0044710 Single-organism metabolic process<br>GO:0006082 Organic acid metabolic process<br>GO:0015909 Long-chain fatty acid transport<br>GO:0000303 Response to superoxide
5	341	GO:0015992 Proton transport<br>GO:0070085 Glycosylation<br>GO:0006812 Cation transport<br>GO:0006068 Ethanol catabolic process<br>GO:0007223 Wnt receptor signaling pathway, calcium modulating pathway
4	223	GO:0006508 Proteolysis<br>GO:0002253 Activation of immune response<br>GO:0046395 Carboxylic acid catabolic process<br>GO:0072376 Protein activation cascade<br>GO:1901565 Organonitrogen compound catabolic process<br>GO:0007596 Blood coagulation
3	459	GO:0006508 Proteolysis<br>GO:0002253 Activation of immune response<br>GO:0046395 Carboxylic acid catabolic process<br>GO:0072376 Protein activation cascade<br>GO:1901565 Organonitrogen compound catabolic process<br>GO:0007596 Blood coagulation
2	693	GO:0044085 Cellular component biogenesis<br>GO:0006396 RNA processing<br>GO:0034660 ncRNA metabolic process<br>GO:0006364 rRNA processing<br>GO:0006725 Cellular aromatic compound metabolic process<br>GO:0044238 primary metabolic process
1	232	GO:0000045 Autophagic vacuole assembly<br>GO:0009267 Cellular response to starvation<br>GO:0002855 Regulation of natural killer cell mediated immune response to tumor cell<br>GO:0007076 Mitotic chromosome condensation<br>GO:0045796 Negative regulation of intestinal cholesterol absorption

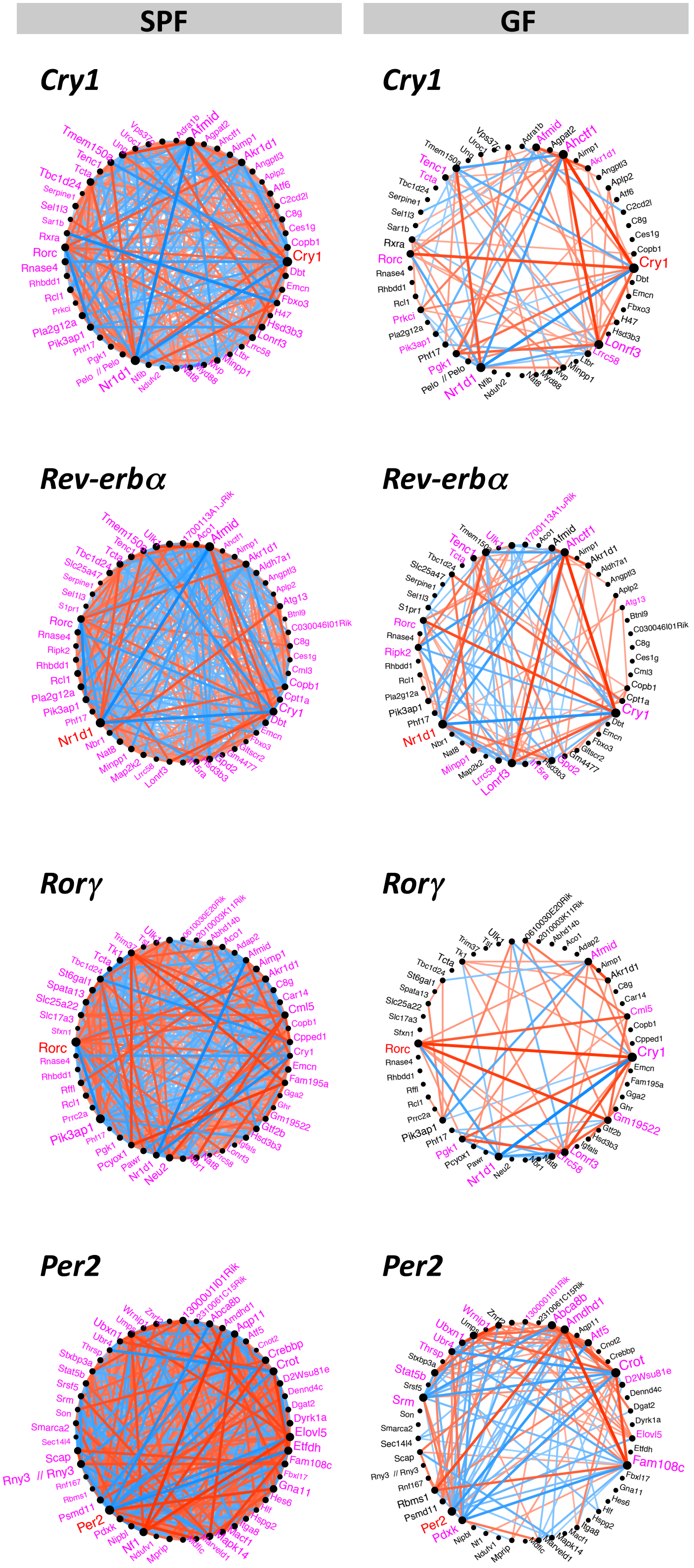
SPF
GF
Cry1
Cry1
Rev-erbα
Rev-erbα
Rorγ
Rorγ
Per2
Per2

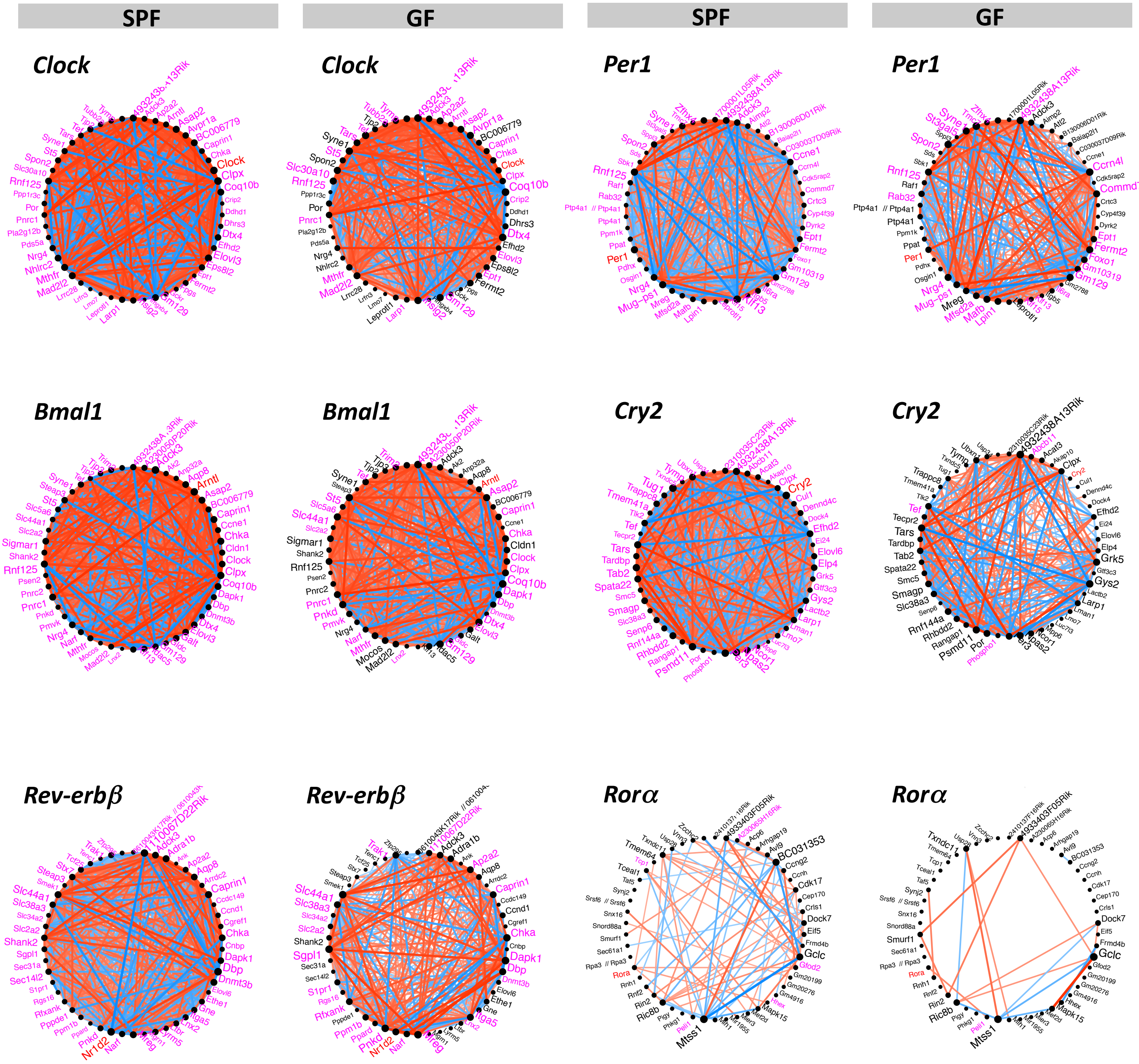

SPF
GF
SPF
GF
Clock
Clock
Per1
Per1
Bmal1
Bmal1
Cry2
Cry2
Rev-erbβ
Rev-erbβ
Rorα
Rorα

Pparα

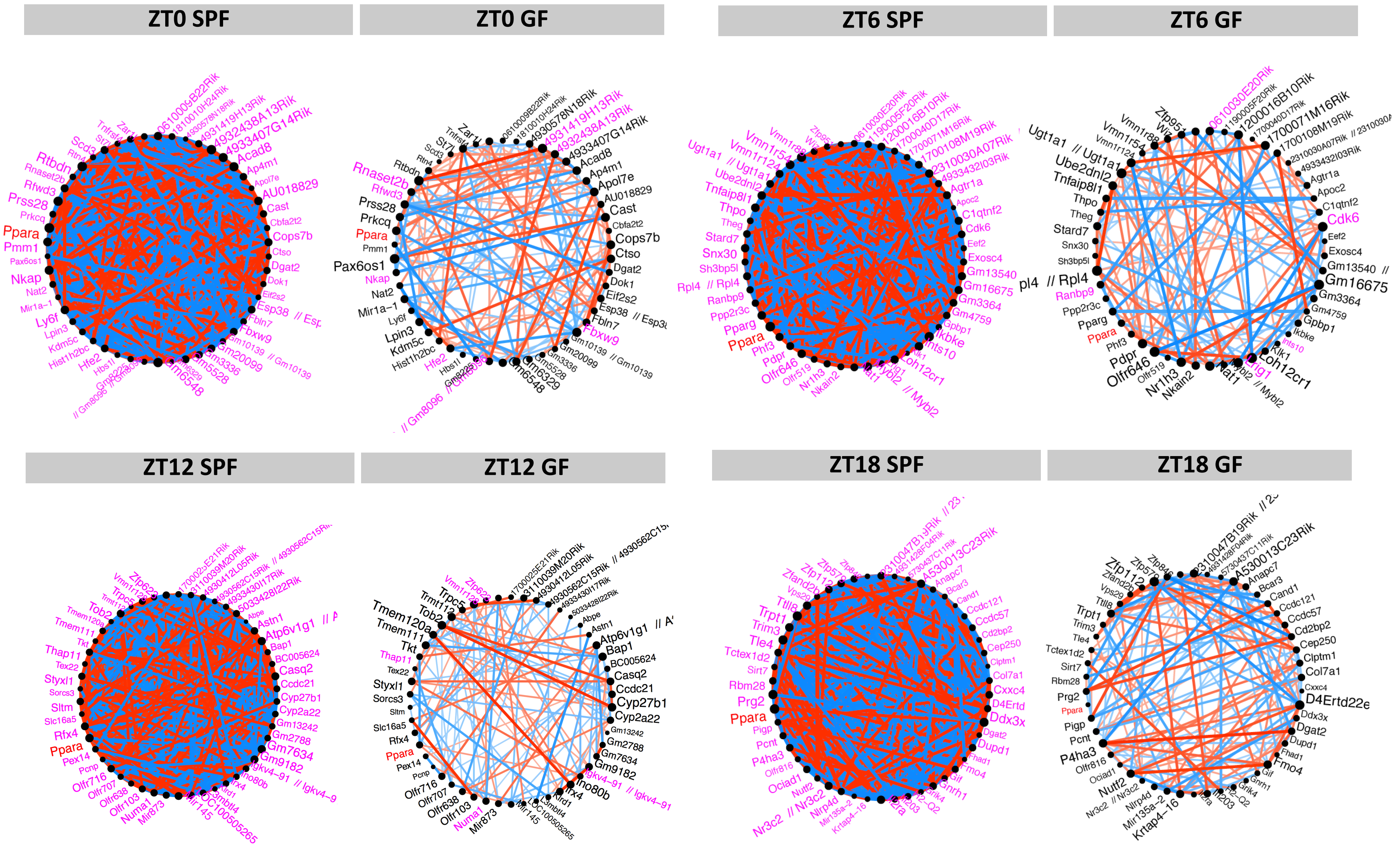

Pparβ/δ

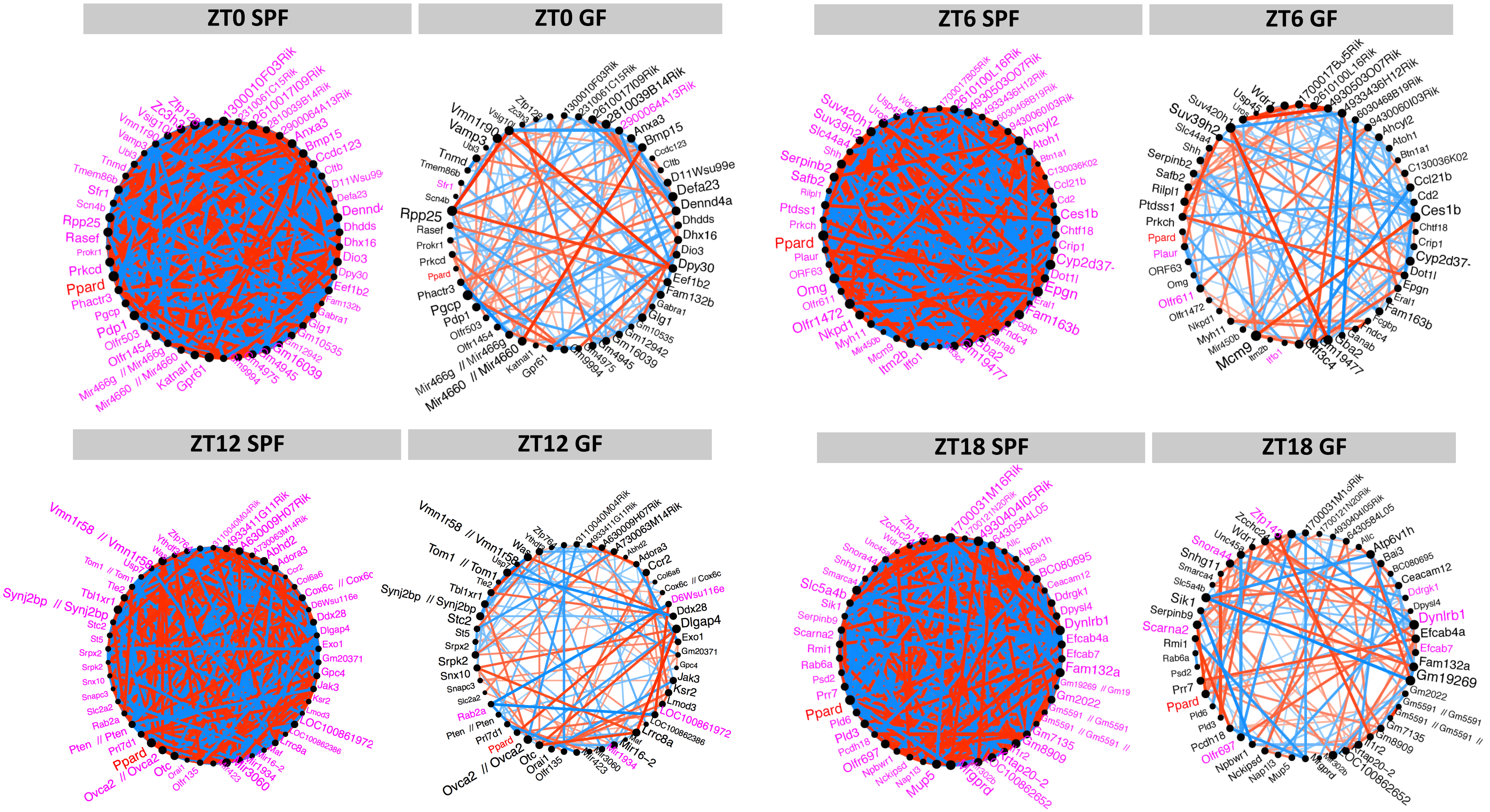

		GF				SPF				
**Metabolites**	**Chemical Shift (ppm)**	*P value*[1]	**ZT0/ ZT6**	**ZT6/ ZT12**	**ZT12/ ZT18**	*P value*[1]	**ZT0/ ZT6**	**ZT6/ ZT12**	**ZT12/ ZT18**	**JTK _Cycle analysis**[2]
**Alanine**	1.48-1.46	0.0034		+		0.0277	-			
**AMP**	8.625	0.0436								
**AMP/ADP/ATP**	8.56; 8.26; 6.14; 4.37	0.0029		+		0.0186			-	5
**Betaine**	3.91; 3.27-3.25	0.0034		-	+	0.0158				
**Glucose**	5.23; 3.90-3.88; 3.86-3.82; 3.78-3.69; 3.54-3.50; 3.48-3.45; 3.40-3.38	0.0008		-		0.0043		-		5
**Glutamate/proline**	2.09-2.08	0.0012	-	+		0.0357				
**Glutathione**	2.98; 2.96-2.92; 2.58-2.52; 2.18-2.15	0.0015	-	+		0.0041	-			5
**Glycerophospho choline**	4.35-4.33; 3.23	0.0191				0.0071			-	
**Glycine**	3.55	0.0013		+						
**Glycogene**	5.43-5.40; 3.63	0.0017				0.0024		-		1
**Inosine**	8.35; 6.11-6.10	0.0071	+			0.0302				5
**Lactate**	4.13-4.09; 1.33-1.31	0.0128	+							
**Lysine**	3.03-3.01; 1.94-1.93; 1.90-1.88; 1.74-1.70; 1.52-1.50	0.0005		+		0.0036	+	+		5
**NAD+**	9.33; 8.83; 8.81; 8.18; 8.16; 6.08-6.07					0.0134		+		
**Ornithine**	3.06-3.04	0.0009		+		0.0205				
**Phenylalanine**	7.36					0.0068		+		
**Proline**	2.05; 2.03-2.02	0.0014		+						5
**Serine**	3.99-3.95	0.0031	-			0.0043	-			4
**Succinate**	2.40	0.0196		+		0.0429				
**Taurine**	3.43-3.42	0.0132			-	0.0320				
**Tyrosine**	7.18					0.0124		+		
**Uridine**	7.89					0.0473			-	
**Valine**	1.05; 0.99-0.98	0.0061		+						

Marker ID	ZT0_var.equal	ZT6_var.equal	ZT12_var.equal	ZT18_var.equal	**ZT0_BH.adj.p**	**ZT6_BH.adj.p**	**ZT12_BH.adj.p**	**ZT18_BH.adj.p**
**HDL cholesterol**	1	1	1	1	**0.00180**	0.09310	0.45913	0.69898
Free Fatty Acid	1	1	1	1	0.74178	0.08878	0.44823	0.23869
**Bilirubin**	1	1	1	1	**0.00008**	0.08878	0.46078	0.13790
**Lactate**	0	1	1	1	**0.00008**	0.34131	0.10911	0.31370
Cholesterol	1	1	1	1	0.37838	0.08878	0.27881	0.73009
FGF21	0	0	0	0	0.32409	0.11900	0.15632	0.32130

Marker ID	**SPF_BH.pvalue**	SPF_JTK.ADJP	SPF_PERIOD	SPF_LAG	SPF_AMP	**GF_BH.pvalue**	GF_JTK.ADJP	GF_PERIOD	GF_LAG	GF_AMP	class
**Bilirubin**	**0.0013**	0.003	18	3	0.7106	0.0854	0.0285	12	9	0.6293	**4**
**FGF21**	**0.0004**	0.0000	24	9	668.7816	0.8946	0.8946	0	0	0,0000	**4**
**FFA**	0.17446	0.0582	18	15	0.1414	**0.00956**	0.0015	24	6	0.1131	**5**
**Lactate**	0.3581	0.15916	12	6	5.2397	**0.0096**	0.0021	18	12	2.0259	**5**
HDL cholesterol	0.8300	0.5913	24	18	0.0919	0.2055	0.1370	12	0	0.1237	6
Cholesterol	0.9472	0.9472	18	6	0.0460	0.1065	0.0592	24	9	0.1662	6

Gene ID	NCBI Refseq	Forward primer (5'-3')	Reverse primer (5'-3')
Bmal1	NM_007489	CAAACTACAAGCCAACATTTCTATCAG	TCGGTCACATCCTACGACAAAC
Car	NM_009803	GCTGCAAGGGCTTCTTCAGA	CCTTCCAGCAAACGGACAGA
Chrebp	NM_021455	ATGACCCCTCACTCAGGGAATA	GATCCAAGGGTCCAGAGCAG
Chrebpa	NM_021455	CGACACTCACCCACCTCTTC	TTGTTCAGCCGGATCTTGTC
Chrebpb	JQ437838	TCTGCAGATCGCGTGGAG	CTTGTCCCGGCATAGCAAC
Cry1	NM_007771	CAGCTGATGTATTTCCCAGGCT	CAATTGAGAGTTTAGTGATGTTCCATTC
Cyp2b10	NM_009999	TTTCTGCCCTTCTCAACAGGAA	ATGGACGTGAAGAAAAGGAACAAC
Cyp3a11	NM_007818	TCACACACACAGTTGTAGGCAGAA	GTTTACGAGTCCCATATCGGTAGAG
Cyp4a14	NM_007822	TCAGTCTATTTCTGGTGCTGTTC	GAGCTCCTTGTCCTTCAGATGGT
Dbp	NM_016974	AAGAAGGCAAGGAAAGTCCA	TGTACCTCCGGCTCCAGTA
Dec2	NM_001271768	GCGAGACGATACCAAGGATAC	TCAGATGTTCGGGCAGTAAA
Fasn	NM_007988	AGTCAGCTATGAAGCAATTGTGGA	CACCCAGACGCCAGTGTTC
Fgf21	NM_020013.4	AAAGCCTCTAGGTTTCTTTGCCA	CCTCAGGATCAAAGTGAGGCG
Lpk	NM_013631	TCGACTCAGAGCCTGTGGC	AGTCGTGCAATGTTCATCCCT
LXRa	NM_013839	GGAGTGTCGACTTCGCAAATG	TCAAGCGGATCTGTTCTTCTGAC
Per1	NM_011065	ACCAGCGTGTCATGATGACATAC	CTCTCCCGGTCTTGCTTCAG
Per2	NM_011066	ATGCTCGCCATCCACAAGA	GCGGAATCGAATGGGAGAAT
Ppara	NM_011144	CCCTGTTTGTGGCTGCTATAATTT	GGGAAGAGGAAGGTGTCATCTG
Pxr	NM_010936	AGAGATCATCCCTCTTCTGCCAC	GATCTGGTCCTCAATAGGCAGGT
Reverba	NM_145434	CAGCTGGTGAAGACATGACGAC	GGGAAGAGGAAGGTGTCATCTG
Reverbb	NM_011584	CGCCATGGAGCTGAACG	GACAAGAGGCAGGGCTGGA
Tbp	NM_013684	ACTTCGTGCAAGAAATGCTGAA	GCAGTTGTCCGTGGCTCTCT
Tef	NM_017376	GCCGAGCTTCGCAAGGA	ACAGGTTACAAGGGCCCGTACT
